# Downregulation of *RCN1* promotes pyroptosis in acute myeloid leukemia cells

**DOI:** 10.1002/1878-0261.13521

**Published:** 2023-09-30

**Authors:** Sisi Deng, Yuming Pan, Na An, Fengyi Chen, Huan Chen, Heng Wang, Xiaojing Xu, Rui Liu, Linlin Yang, Xiaomei Wang, Xin Du, Qiaoxia Zhang

**Affiliations:** ^1^ Shenzhen Bone Marrow Transplantation Public Service Platform, Shenzhen Institute of Hematology, Shenzhen Second People's Hospital First Affiliated Hospital of Shenzhen University, Shenzhen University Health Sciences Center China; ^2^ Department of Physiology, School of Basic Medical Sciences, International Cancer Center Shenzhen University Health Sciences Center China; ^3^ Department of Hematology Shenzhen Longhua District Central Hospital China; ^4^ China National GeneBank, BGI‐Shenzhen China

**Keywords:** AML, caspase‐1, GSDMD, IFN‐1, pyroptosis, RCN1

## Abstract

Reticulocalbin‐1 (RCN1) is expressed aberrantly and at a high level in various tumors, including acute myeloid leukemia (AML), yet its impact on AML remains unclear. In this study, we demonstrate that *RCN1* knockdown significantly suppresses the viability of bone marrow mononuclear cells (BMMNCs) from AML patients but does not affect the viability of granulocyte colony‐stimulating factor (G‐CSF)‐mobilized peripheral blood stem cells (PBSCs) from healthy donors *in vitro*. Downregulation of *RCN1* also reduces the viability of AML cell lines. Further studies showed that the *RCN1* knockdown upregulates type I interferon (IFN‐1) expression and promotes AML cell pyroptosis through caspase‐1 and gasdermin D (GSDMD) signaling. Deletion of the mouse *Rcn1* gene inhibits the viability of mouse AML cell lines but not the hematopoiesis of mouse bone marrow. In addition, *RCN1* downregulation in human AML cells significantly inhibited tumor growth in the NSG mouse xenograft model. Taken together, our results suggest that RCN1 may be a potential target for AML therapy.

## Graphical abstract

This study showed that *RCN1* downregulation upregulates type I interferon (IFN‐1) by activating the STING pathway. Consequently, this results in an increased expression of cleaved‐caspase‐1 and IL‐1β, which triggers pyroptosis in AML cells via gasdermin D (GSDMD) signaling. These findings provide potential targets for AML therapy.

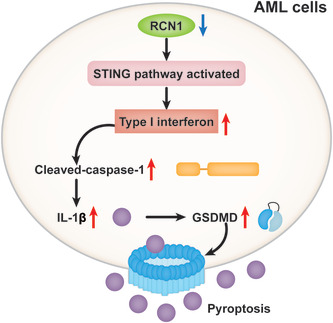

**Abbreviations**
ALDH1: acetaldehyde dehydrogenase type 1
AML: acute myeloid leukemia
ASC: apoptosis‐associated speck‐like protein that contains a carboxyl‐terminal CARD
BEAS‐2B: human normal lung epithelial cells
BMMNCs: bone marrow mononuclear cells
Cas9: CRISPR‐associated protein 9
CBC: complete blood count
CFUs: colony‐forming units
CLP: common lymphoid progenitor
CMP: common myeloid progenitors
CNGB: China National Gene Bank
CRISPR: clustered regularly interspaced short palindromic repeats
DSF: disulfiram
EHD2: EH domain‐containing protein 2
ER: endoplasmic reticulum
FAB: French‐American‐British
G‐CSF: granulocyte colony‐stimulating factor
GEPIA2: gene expression profiling interactive analysis 2
GMP: granulocyte–macrophage progenitors
GSDMD: gasdermin D
GTEx: genotype‐tissue expression
HaCaT: human immortalized keratinocytes
HCT: hematocrit
HFF‐1: human foreskin fibroblasts
HGB: hemoglobin
HMAs: hypomethylating agents
HPC: hematopoietic progenitor cell
HSCs: hematopoietic stem cells
HSCT: hematopoietic stem cell transplant
HVGs: highly variable genes
IFN‐1: type I interferon
IFNAR: interferon receptor
IN‐1: IFN alpha‐IFNAR‐IN‐1 hydrochloride
ISGs: interferon‐stimulated genes
LMPP: lymphoid‐primed multipotent progenitor
LPS: lipopolysaccharide
MEP: megakaryocyte erythroid progenitors
MKP: megakaryocyte progenitors
MPP: multipotent progenitor
MPV: mean platelet volume
MyD88‐I: inhibitors for MyD88
N‐LECs: lymphatic endothelial cells
PBSCs: peripheral blood stem cells
PDW: platelet distribution width
PIPC: polyinosinic acid·polycytidylic acid
RBC: red blood cell count
RCN1: reticulocalbin‐1
SAHA: suberonylanilide hydroxamic acid
ScRNA: single‐cell RNA
STING‐I: inhibitors for STING
TCGA: The Cancer Genome Atlas
T‐LECs: lymphatic endothelial cells
TPA: 12‐o‐tetradecanoylphorbol‐13‐acetate
UMI: unique molecular identifier
UPR: unfolded protein response
ZIC1: ZIC family member 1

## 1 Introduction

Acute myeloid leukemia (AML) is a clonal malignant disease characterized by suppression of hematopoiesis and halted differentiation in bone marrow precursors due to genetic abnormalities [1, 2, 3, 4, 5]. Traditional treatments for AML patients include hypomethylating agents (HMAs), hematopoietic stem cell transplant (HSCT), and chemotherapy, but these therapies have demonstrated poor therapeutic outcomes [6, 7, 8]. Recent advances in medicine, such as BCL‐2 inhibitors, FLT3 inhibitors, and IDH inhibitors [9, 10, 11, 12], have improved patient outcomes. However, the high rate of relapse remains a clinical challenge, affecting both younger patients (40%) and the vast majority of elderly patients [6, 13, 14, 15]. Additionally, IDH inhibitors can cause differentiation syndrome as a targeted side effect [16], while BCL‐2 inhibitors exhibit pan‐activity [17], and FLT3 inhibitors are associated with highly toxic side effects [18]. Therefore, it is imperative to identify novel molecular targets to address these issues.

Reticulocalbin‐1 (RCN1), an endoplasmic reticulum protein, plays a crucial role in calcium homeostasis and inhibits ER stress‐induced apoptosis [19, 20]. In multiple cancers, such as glioblastoma, non‐small cell lung cancer, renal cell carcinoma, hepatocellular carcinoma, and oral squamous cell carcinoma, the overexpression of *RCN1* has been observed indicating its involvement in tumorigenesis and invasion [21, 22, 23, 24, 25]. High levels of RCN1 expression have been associated with sorafenib resistance in hepatocellular carcinoma and doxorubicin resistance in uterine cancer cells [23, 26]. Conversely, downregulation of RCN1 has been found to inhibit cell proliferation and promote cell death by activating the AKT and PTEN pathways in prostate cancer cells [27]. These findings suggest that RCN1 may be a potential therapeutic target. Our data show that RCN1 is highly expressed in AML patients, but its effect on AML and the mechanism behind it remain to be determined.

Type I interferon (IFN‐1) is a critical regulator of the immune system. When the cell detects microbial components like lipopolysaccharide (LPS) or external nucleic acid sequences, it activates the production of IFN‐1. IFN‐1 then binds to the interferon receptor (IFNAR) on the cell membrane, which leads to the activation of interferon‐stimulated genes (ISGs) [28, 29, 30, 31, 32, 33, 34]. STING agonists, including cyclic dinucleotides and derivatives, vadimezan, and small‐molecule agonists, have entered clinical trials as anticancer medicines, demonstrating the potential for a new field of tumor immunotherapy [35, 36, 37, 38, 39, 40, 41, 42, 43]. Furthermore, IFN‐1 has been utilized to prevent leukemia relapse following allogeneic transplantation [44, 45].

Our results indicate that knockdown of RCN1 in human AML cell lines upregulates IFN‐1, which triggers cell pyroptosis via caspase‐1 and gasdermin D (GSDMD) signaling. We observed a decrease in cell viability of AML cells both *in vivo* and *in vitro* following *RCN1* knockdown, providing a new target for AML therapy.

## 2 Materials and methods

### 2.1 Patient samples

Healthy donor samples were obtained from G‐CSF‐mobilized peripheral blood stem cells (PBSCs) from healthy donors. AML patient samples were obtained from the bone marrow mononuclear cells (BMMNCs) of AML patients. Written informed consent was obtained from all participants. The samples were collected from December 2016 to November 2017 at Shenzhen Second People's Hospital. This study complies with the Declaration of Helsinki and was approved by the medical ethics committee of Shenzhen Second People's Hospital (Approval number: 2016121603).

### 2.2 Cell culture and transfection

MOLM‐13 cells (RRID: CVCL_2119), NB4 cells (RRID: CVCL_0005), OCI/AML3 cells (RRID: CVCL_1844), THP‐1 cells (RRID: CVCL_0006), and 293T/17 cells (RRID: CVCL_1926) were purchased from the National Collection of Authenticated Cell Cultures (China). BEAS‐2B cells (RRID: CVCL_0168), HaCaT cells (RRID: CVCL_0038), HFF‐1 cells (RRID: CVCL_3285), HMEC‐1 cells (RRID: CVCL_0307), and MRC‐5 cells (RRID: CVCL_0440) were purchased from Shanghai Genechem Company (Shanghai, China). These cell lines have been authenticated in the past 3 years using short tandem repeat analysis. All the cells were cultured in 5% CO_2_ and maintained *in vitro* in Dulbecco's modified Eagle's medium or RPMI 1640 medium supplemented with 10% heat‐inactivated fetal bovine serum (FBS, 35‐081‐CV; Corning, Manassas, VA, USA), and 100 U·mL^−1^ penicillin–streptomycin (15140122; Gibco, New York, NY, USA). Two specific sgRNA (sgRNA‐1 and sgRNA‐2, Table S1) lines were designed and then cloned into the Puc57‐sample plasmid and co‐transfected into MOLM‐13 cells with hCas9 and EGFP fusion protein expression vector pHS‐CR028 by electroporation; NB4 cells, OCI/AML3 cells, THP‐1 cells, 293T/17 cells, BEAS‐2B cells, HaCaT cells, HFF cells, HMEC‐1 cells, and MRC‐5 cells were infected by lentivirus; sh‐*RCN1* was designed and cloned into the GV248 vector. Cells were treated with 1–2 μg·mL^−1^ puromycin (58‐58‐2; Invivogen, San Diego, CA, USA) 24 h after being transfected with lentivirus. NIH/3T3 cells, J774A.1 cells, and Raw264.7 cells were transfected with siRNA (Si‐Rcn1‐1 and Si‐Rcn1‐2). In this study, the cells used in all experiments were mycoplasma‐free.

### 2.3 Cell viability

Cell viability was evaluated by the CCK solution (N31213; TransGen Biotech, Beijing, China). Treated cells were incubated with 10 μL of CCK‐8 (5 mg·mL^−1^) for 1 h. Then, cell response was determined on a plate reader (Tristar 2 S LB 942 multimode reader, Berthold Technologies, Bad Wildbad, Germany).

### 2.4 Western blot

Samples were boiled to denature proteins and separated in 12% mini PROTEAN TGX Precast Protein Gels (4561045; Bio‐rad, Hercules, CA, USA). Proteins were then transferred to 0.22 μm PVDF membrane with a standard wet transfer system at 100 V for 1 h. Antibodies were diluted in 5% BSA at the appropriate concentration (according to the manufacturers' instructions). Membranes were blocked with 5% skim milk for 1 h and washed away with TBST (50 mm Tris pH 8.0, 150 mm NaCl, 0.1% Tween 20) twice and then incubated with primary antibodies overnight at 4 °C on a shaker. Membranes were washed with TBST five times and incubated with HRP‐linked secondary antibodies for 1 h at room temperature on a shaker. After five washes, membranes were developed with Merck Millipore WBULS0100 Immobilon® ECL HRP Substrate (WBULS0100; Millipore, Billerica, MA, USA). Antibodies were used at the following dilutions in 5% BSA: Anti‐RCN1 (ab1989996; Abcam, Cambridge, UK), cleaved‐caspase‐1 (YC0002; Immunoway, Plano, TX, USA), GMDSD (A20197; ABclone, Woburn, MA, USA), Anti‐Lysozyme (ab108508; Abcam), Anti‐ISG15 (ab133346; Abcam), OAS1 Antibody (abs137237; Absin, Shanghai, China), Anti‐OAS3 antibody (ab154270; Abcam), Anti‐OAS2 (G‐9) (sc‐271117; Santa Cruz Biotechnology, Santa Cruz, CA, USA), Anti‐IFN‐α (sc‐80996; Santa Cruz Biotechnology), Anti‐IFN‐β (ab176343; Abcam), Beclin (ab207612; Abcam), Anti‐β‐actin (4970; Cell Signaling Technology, Danvers, MA, USA), Anti‐GADPH (abs830030a; Absin), HRP‐Goat Anti‐Mouse IgG (RS0001; Immunoway), HRP‐Goat Anti‐Rabbit IgG (RS0002; Immunoway), Recombinant Anti‐IL‐1 beta antibody (ab216995; Abcam), CHOP (L63F7) Mouse mAb (2895T; Cell Signaling Technology), PERK (D11A8) Rabbit mAb (5683T; Cell Signaling Technology), BiP (C50B12) Rabbit mAb (3177T; Cell Signaling Technology), and PERK (phospho Thr981) Polyclonal Antibody (YP1055; Immunoway). Antibody concentration according to the instructions.

### 2.5 RNA extraction, reverse‐transcription and quantitative PCR

Reverse‐transcription and quantitative PCR‐RNA were isolated using the Steady Pure Universal RNA Extraction Kit II (AG21022; Accurate Biology, Hunan, China). Reverse transcription of 1 μg RNA was performed using the Evo M‐MLV Plus cDNA Synthesis Kit (AG11615; Accurate Biology). Quantitative PCR was carried out in triplicate with target‐specific primers using Novo Start®SYBR qPCR Super Mix Plus and quantitated using the 7300plus Real‐Time PCR System (Applied Biosystem, Waltham, MA, USA).

### 2.6 ScRNA‐seq data processing and quality control

ScRNA‐seq data processing and analysis according to a previously published paper by Shi et al. [46]. Single‐cell RNA (ScRNA) sequencing libraries were constructed using DNelabC4 according to the manufacturer's instructions. The libraries were quantified using a Qubit ssDNA analysis kit (Thermo Fisher Scientific, Waltham, MA, USA) and sequenced using the DIPSEQ T1 sequencer of the China National Gene Bank (CNGB). High‐quality scRNA sequencing data with valid barcodes were aligned to the genome of human reference genome (GRCh38) through star [47], and the unique molecular identifier (UMI) count matrix was generated using pisa (version 1.10.2) (https://github.com/shiquan/PISA). seurat v4.0.1 (https://github.com/satijalab/seurat) was applied for clustering analysis. First, cells with mitochondrial gene counts greater than 10% were excluded, as were cells expressing fewer than 400 or more than 4500 genes. Doublet Finder (version 2.0.3) [48] was employed to remove doublets with assuming 5% doublet formation rate. Then, the filtered data were normalized and scaled by the ‘Normalize Data’ and ‘Scale Data’ functions using default parameters, respectively. The top 2000 highly variable genes (HVGs) for each library were used for further processing. Next, all the datasets were integrated using the ‘Find Integration Anchors’ and ‘Integrate Data’ functions in Seurat. Finally, we conducted dimension reduction for the scaled merged dataset by PCA analysis. The first 30 principal components were used to construct a K‐nearest neighbor graph through the ‘Find Neighbors’ function, and the cell clusters were assigned through the ‘Find Clusters’ function. The visualization was shown by the UMAP. The ‘Find All Markers’ function in Seurat was used to identify cluster‐specific marker genes (thresh.use = 0.25, min.pct = 0.25, only.pos = TRUE).

### 2.7 Mice

All mice were bred and maintained under specific pathogen‐free conditions at the animal facility of Topbiotech Company (Shenzhen, China). All mice were provided with adequate housing, nutrition, water, handling, ventilation, sanitation, and veterinary care. Animal protocols were consistent with the National Institutes of Health guidelines. Animal experiments were performed under study approved by the Topbiotech Company (Animal license number: SYXK(Yue)2020‐0230). Floxed *Rcn1* mice were crossed with *Mx1‐cre* and *CMV‐cre* mice to obtain conditional *Rcn1*‐deficient mice. The *Rcn1* ^fl/fl^ mice (C57BL/6) and Mx1‐Cre mice (B6.Cg‐Tg (*Mx1‐cre*) 1Cgn/J) were purchased from Shanghai Model Organisms (Shanghai, China), and the *CMV‐cre* (C001055) mice were purchased from Cyagen Biosciences (Suzhou, China). The *Rcn1* gene was changed by flox employing homologous recombination of fertilized eggs according to the principle of homologous recombination. Mice were 4–10 weeks of age when they crossed with each other. Polyinosinic Acid·Polycytidylic Acid (pIpC) was purchased from Merck (Rahway, NJ, USA; 528906‐10MG). Six‐ to –ten‐week‐old mice were treated with 10 μg PIPC every other day for five times. Four‐ to six‐week‐old female NCG (NOD‐Prkdcem26Il2rgem26/Gpt) mice were purchased from Gempharmatech (Nanjing, China). Four‐ to six‐week‐old female NSG mice were purchased from Shanghai Model Organisms. Tumor volumes were measured with a caliper every 4 days by length (*a*), width (*b*), and height (*c*) and calculated as tumor volume = *abc*/2.

### 2.8 Flow cytometry

Six‐ to ten‐week‐old mice were treated with PIPC, and bone marrow cells from mice were isolated from tibias and femurs and transferred to a 100‐mm sterile culture dish at the indicated times. Cells were washed with 1 mL FACS buffer and spun down at 500 ***g*** for 5 min, followed by staining with antibodies in FACS buffer (2% FBS, 0.03% NaN_2_ in PBS) in the dark at 4 °C for 30 min. Cells were washed with 2 mL FACS buffer and spun down at 500 ***g*** for 5 min. Flow cytometry analysis was performed on Novios 10 colors (Beckman Coulter, Brea, CA, USA) instruments. Flow cytometry data were analyzed with flowjo software 10 (BD Biosciences, San Jose, CA, USA). Bone marrow cells are classified into the following categories: hematopoietic stem cells (HSC, CD150^+^CD48^−^LSK), multipotent progenitor (MPP, CD150^−^CD48^−^LSK), megakaryocyte progenitors (MKP, Lin^−^ Sca1^−^cKit^+^CD150^+^CD41^+^), megakaryocyte erythroid progenitors (MEP, CD34^−^FcγR^−^Lin^−^Sca1^−^cKit^+^), common myeloid progenitors (CMP, CD34^+^FcγR^−^Lin^−^Sca1^−^cKit^+^), granulocyte–macrophage progenitors (GMP, CD34^+^FcγR^+^Lin^−^Sca1^−^cKit^+^), LSK cells (Lin^−^Sca1^+^cKit^+^), hematopoietic progenitor cell (HPC, Lin^−^Sca1^−^cKit^+^), common lymphoid progenitor (CLP, Lin^−^Sca1^low^cKit^low^Flt3^+^IL7Rα^+^), and lymphoid‐primed multipotent progenitor (LMPP, Lin^−^ Sca1^+^ cKit^+^ Flt3^+^).

### 2.9 Flow antibody

LIVE/DEAD FIX AQUA (L34966) was purchased from Thermo/Invitrogen (Waltham, MA, USA). The following flow antibodies were purchased from Miltenyi Biotech (Bergisch Gladbach, Germany): anti‐CD5‐APC‐Vio770 (REA421), anti‐Mac (CD11b)‐APC‐Vio 770 (REA592), anti‐CD45R (B220)‐APC‐Vio 770 (REA755), anti‐7‐4 (Ly‐6B.2)‐APC‐Vio770 (REA115), anti‐Gr1‐APC‐Vio 770 (REA526), anti‐Ter‐119‐APC‐Vio770 (REA847), anti‐cKit (CD117)‐APC (REA791), anti‐Sca1‐PE‐Vio 700 (REA422), anti‐CD150‐PE (REA299), anti‐CD48‐Vio Bright 515 (REA1238), anti‐CD41‐VioBlue (REA1194), anti‐CD11b‐VioBright FITC (REA592), anti‐F4/80‐PE (REA126), anti‐CD71‐PE (REA627), anti‐CD4‐PE‐Vio770 (REA1211), anti‐CD8‐APC (REA601), anti‐IgM‐VioBright 515 (REA979), anti‐CD34‐FITC (REA383), anti‐FcγR (CD16/CD32)‐PE (REA377), anti‐Sca1‐VioBright FITC (REA422), anti‐Flt3 (CD135)‐PE‐Vio700 (REA779), and anti‐IL7R (CD127)‐PE (REA680).

### 2.10 Inhibitor assay

NB4 cells or OCI/AML‐3 cells were transfected with lentivirus for 24 h and then added 1–2 μg·mL^−1^ puromycin with or without GSDMD inhibitor (Disulfiram, DSF, HY‐B0240; MedChemExpress, Princeton, NJ, USA), IFN inhibitor (IFN alpha‐IFNAR‐IN‐1 hydrochloride, IN‐1, HY‐12836A; MedChemExpress), STING inhibitor (H151, STING‐I, HY‐112693; MedChemExpress), and MyD88 inhibitor (T6167923, Myd88‐I, HY‐19744; MedChemExpress). Cleaved‐caspase‐1 was detected by the FLICA® 660 Caspase‐1 Assay Kit (9122; Immunochemistry, Bloomington, MN, USA), and cell death was tested by the Annexin V‐PE Apoptosis Detection Kit (559763; BD Pharmingen, San Diego, CA, USA) through flow cytometry.

### 2.11 Colony‐forming unit assay

The equivalent of 3 × 10^4^ bone marrow cells were resuspended to a final volume of 1.5 mL with methylcellulose medium (M3434, MethoCult™ GF; Stemcell Technologies, Vancouver, BC, Canada) and plated in 6‐well plates. After incubating at 37 °C in 5% CO_2_ for 6 days, colony‐forming units (CFUs) were counted and photographed under an Automated CFU Assay Reader (STEMvision™; Stemcell Technologies).

### 2.12 Primary cell culture

Normal samples were derived from G‐CSF‐mobilized PBSCs from healthy donors. AML patient samples were derived from the BMMNCs of AML patients. Then, samples were cultured in GMP SCGM medium (20802‐0500; Cell Genix, Freiburg, Germany) with human recombinant IL‐3 (78194, 60 ng·mL^−1^; Stemcell Technologies), human TPO (GMP‐CJ95, 100 ng·mL^−1^; Novoprotein, Suzhou, China), human SCF (78062.2, 300 ng·mL^−1^; Stemcell Technologies), and human Flt3‐L (300‐19‐100, 300 ng·mL^−1^; Peprotech, Rocky Hill, NJ, USA) and plated in 12‐well plate with 2 × 10^5^ cells/well. Then, primary cells were transfected with lentivirus and centrifuged for 1–2 h, 450 × ***g***, MOI = 50. After 14 h, the culture medium was replaced. After 24 h, the second transfection was performed, and then 1 μg·mL^−1^ puromycin was added for 5 days.

### 2.13 Statistical analysis

The data are presented as the mean ± SEM or mean ± SD. The mean difference between the control and treated groups was determined using Student's *t*‐tests with Welch's correction for unequal variance. Two‐way ANOVA was used to analyze the tumor growth. A value of *P* < 0.05 was considered statistically significant (*P < 0.05, **P < 0.01, ***P < 0.001 and ****P < 0.0001).

## 3 Results

### 3.1 The *RCN1* gene is highly expressed in acute myeloid leukemia

**Fig. 1:**
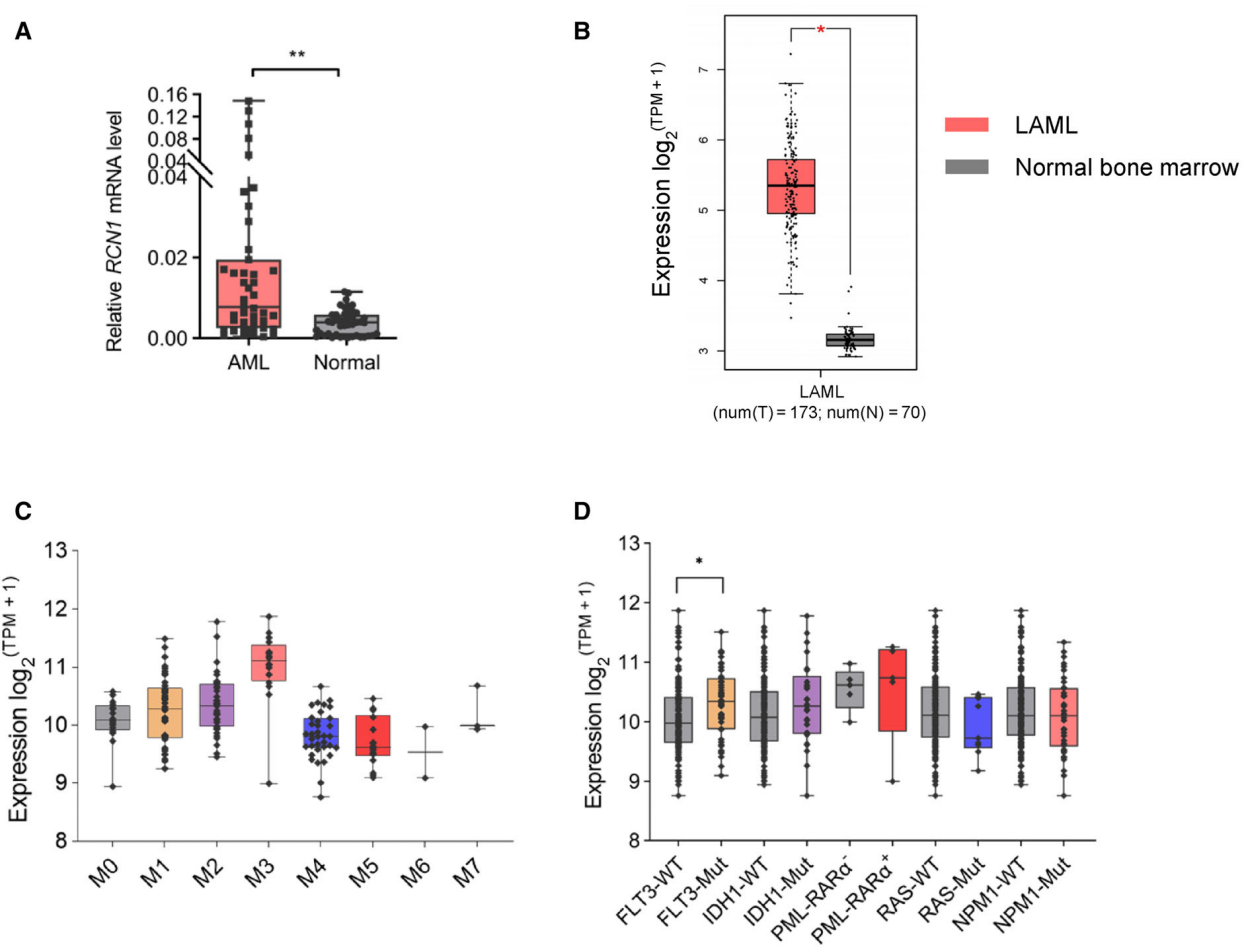
*RCN1* gene is highly expressed in acute myeloid leukemia (AML). (A) Relatively high *RCN1* mRNA level in the bone marrow mononuclear cells (BMMNCs) of AML patients (*n* = 43) compared with G‐CSF‐mobilized peripheral blood mononuclear cells from healthy donors (*n* = 45); values are shown as individual points with the mean ± SEM. (B) *RCN1* expression in AML (LAML) compared with normal bone marrow, data from the TCGA and GTEx datasets. Values are shown as individual points with the mean ± SD (red asterisk), *P < 0.05, as determined by the unpaired two‐tailed Student's *t*‐test. (C) The box plot shows the expression level of *RCN1* in 171 AML patients with various FAB types in the TCGA and GTEx datasets. M0 (*n* = 16), M1 (*n* = 42), M2 (*n* = 39), M3 (*n* = 16), M4 (*n* = 35), M5 (*n* = 18), M6 (*n* = 2), and M7 (*n* = 3). (D) The box plot shows the expression level of *RCN1* in 171 AML patients with various molecular mutations in the TCGA and GTEx datasets. *N* indicates the number of samples. Values are shown as individual points with the mean ± SD. *P < 0.05, **P < 0.01, as determined by the unpaired two‐tailed Student's *t*‐test.

To explore new therapeutic targets for AML, we conducted a transcriptome analysis on BMMNCs from five AML patients and granulocyte colony‐stimulating factor (G‐CSF)‐mobilized PBSCs from five healthy donors (NCBI database SRA accession: PRJNA576718). We identified hundreds of differentially expressed genes, and the *RCN1* gene ranked second on the list. Table S2 shows the top 20 genes that were abnormally expressed. To further confirm the high expression of RCN1 in AML, we assessed *RCN1* mRNA levels by quantitative polymerase chain reaction (qPCR) in 43 BMMNC samples from AML patients and 45 G‐CSF‐mobilized PBSC samples from healthy donors. Participant information is listed in part in Table S3. We observed that the *RCN1* mRNA levels of AML patients were about five times higher than those in healthy donors (Fig. 1A). Furthermore, we utilized the gene expression profiling interactive analysis 2 (GEPIA2) online tool to analyze *RCN1* gene expression based on The Cancer Genome Atlas (TCGA) and Genotype‐Tissue Expression (GTEx) datasets. The mRNA levels of RCN1 in LAML (acute myeloid leukemia) patients were found to be approximately five times greater than those observed in normal bone marrow (Fig. 1B). These results are in line with the findings obtained from our qPCR data.

Acute myeloid leukemia (AML) is a disease that exhibits high heterogeneity. We explored the heterogeneity of primary AML cells by analyzing the morphological typology. To categorize AML subtypes based on morphological characteristics, The French‐American‐British (FAB) typology was employed [49], which resulted in the identification of eight distinct subtypes, ranging from M0 to M7. We tested the expression levels of *RCN1* in various subsets of AML patients obtained from the TCGA and GTEx datasets. The results showed that *RCN1* was relatively more highly expressed in the M3, M2, and M1 subgroups (Fig. 1C). Molecular studies have identified AML subsets characterized by driver mutational events, such as NPM1, FLT3–ITD, and IDH1 mutations [50]. We undertook an analysis of *RCN1* expression in various molecular subsets of AML from the TCGA and GTEx datasets. Patients with AML were categorized into groups based on the presence of FLT3, IDH1, RAS, and NPM1 mutations. The results revealed that, among these groups, only those with FLT3 mutations showed significantly higher levels of *RCN1* expression compared with the wild‐type group (Fig. 1D). Those results suggested that the high expression of *RCN1* may be associated with the FLT3 mutation.

**Fig. 2:**
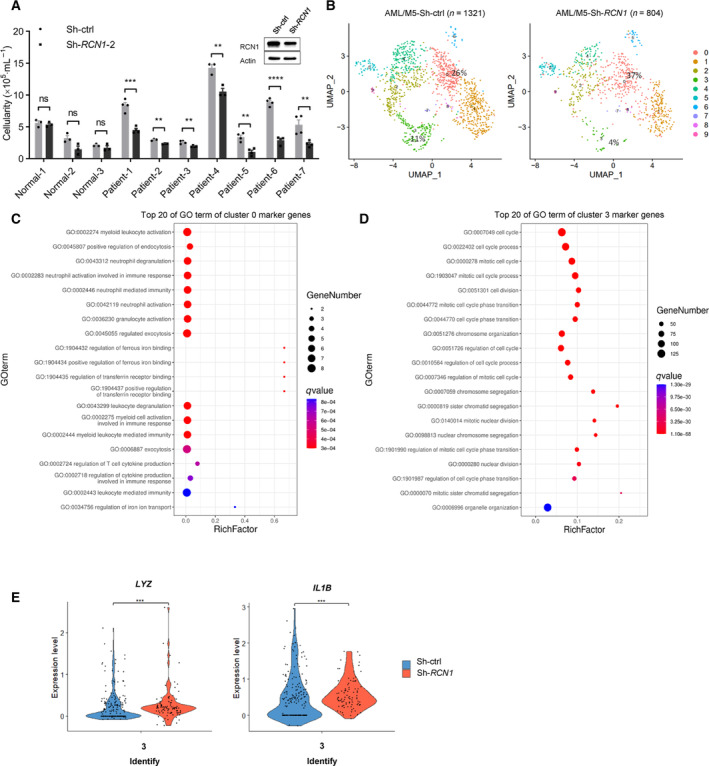
Downregulation of RCN1 significantly inhibits the viability of primary AML cells but not normal hematopoietic cells. (A) The cellularity of the BMMCs from AML patients and the G‐CSF‐mobilized PBMCs from healthy donors 6 days after being transfected with lentivirus sh‐ctrl (Normal‐1–3, Patient‐2, and Patient‐4, *n* = 3; Patient‐1, Patient‐3, and Patient‐5–8, *n* = 4) or sh‐*RCN1* (Normal‐1–3, Patient‐2, and Patient‐4, *n* = 3; Patient‐1, Patient‐3, and Patient‐5–8, *n* = 4). *N* indicates the technical duplicates within one experiment of three independent replicates. Knockdown was confirmed by western blot. (B–D) Single‐cell transcriptome profiling of BMMCs obtained from AML patients transfected with the lentivirus sh‐ctrl or sh‐*RCN1* was performed. UMAP shows single‐cell patterns in a 2D space (B); representative GO terms enriched in marker genes of Cluster 0 (C) and Cluster 3 (D) are shown. (E) The gene expression of *LYZ* and *IL1B* in Cluster 3 was analyzed. One of the two independent experiments is shown. Data are presented as the mean ± SEM. ^ns^ *P* > 0.05, **P < 0.01, ***P < 0.001, ****P < 0.0001, as determined by unpaired two‐tailed Student's *t*‐test.

To investigate the effect of RCN1 on primary AML cells from patients, we knocked down the *RCN1* gene by lentivirus. Seven cases of BMMNCs from AML patients and three cases of G‐CSF‐mobilized PBSCs from healthy donors were cultured *in vitro* and treated with the lentivirus Sh‐ctrl or Sh‐*RCN1*. The cellularity of BMMNCs from AML patients decreased significantly 6 days after being infected with the lentivirus Sh‐*RCN1*, but the PBSCs from healthy donors showed no significant change (Fig. 2A). Next, we performed single‐cell RNA sequencing of another case of primary AML cells from an AML‐M5 patient with *RCN1* knockdown (AML/M5‐sh‐*RCN1*) and control (AML/M5‐sh‐Ctrl). After stringent quality control and filtering using multiple criteria, the transcriptomes of 1321 and 804 single cells (AML/M5‐sh‐ctrl and AML/M5‐sh‐*RCN1*) were acquired, and a mean of 2865 and 2782 genes were detected per cell, respectively (Fig. 2B and Fig. S1A–C). We performed cell clustering and assigned all cells to 10 subgroups, labeled as Clusters 0–9. Cluster 0 and Cluster 3 showed the greatest difference in proportion between the two samples. In the AML/M5‐sh‐*RCN1* group, Cluster 0 accounted for 37% of the total, compared to 26% in the AML/M5‐sh‐ctrl group, while Cluster 3 accounted for 4% of the total, down from 11% in the control group (Fig. 2B). The proportion of clusters per sample is listed in Table S4. Gene ontology analysis of marker genes in Cluster 0 demonstrated that enrichment of genes related to leukocyte function (Fig. 2C), and Cluster 3 related to cell viability (Fig. 2D). The gene number in the enrichment result of Cluster 0 from single‐cell RNA‐seq is too small. Therefore, we focus our attention on the relevant genes in Cluster 3. When we analyzed the gene expression in Cluster 3, the results showed that *LYZ* and *IL‐1B*, two ISG genes, were upregulated after treatment with Sh‐*RCN1* (Fig. 2E). These findings suggest that the downregulation of RCN1, leading to inhibition of primary AML cell viability, may be associated with the upregulation of ISGs.

### 3.2 Decrease in RCN1 inhibits the viability of human AML cell lines

**Fig. 3:**
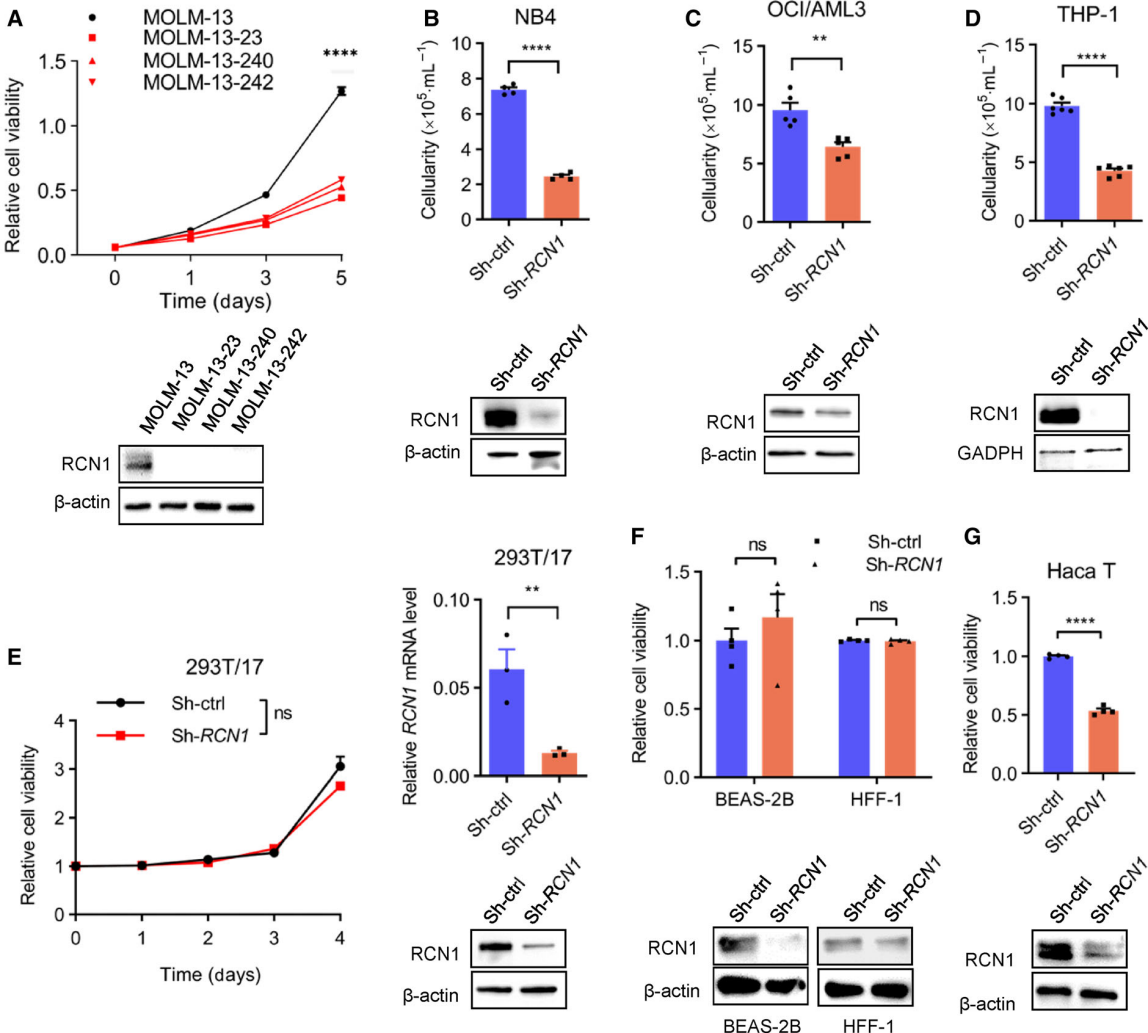
Reduction of RCN1 inhibits the viability of human AML cells. (A) Relative cell viability of the AML cell line MOLM‐13 (*n* = 3), and 3 RCN1 knockout cell clones MOLM‐13‐23 (*n* = 3), MOLM‐13‐240 (*n* = 3), and MOLM‐13‐242 (*n* = 3) were detected by the CCK‐8 assay. RCN1 protein levels of these cells were detected by western blot. One of the two independent experiments is shown. (B–D) The relative cell viability of the AML cell lines NB4 (*n* = 4), OCI/AML3 (*n* = 5), and THP‐1 (*n* = 6) was measured by cell counting using trypan blue 7 days after transfection with lentivirus sh‐ctrl or sh‐*RCN1*; RCN1 reduction was confirmed by western blot analysis. One of the three independent experiments is shown. (E–G) Relative cell viability of immortalized nontumor cells 293T/17 (*n* = 6) (E, left), BEAS‐2B (*n* = 4) (F), HFF‐1 (*n* = 4) (F), and Haca T (*n* = 4) (G) was detected by the CCK‐8 assay 10 days after being transfected with lentivirus sh‐ctrl or sh‐*RCN1*. Relative mRNA levels of *RCN1* in 293 T/17 cells were confirmed by qPCR 4 days after being transfected with lentivirus sh‐ctrl (*n* = 3) or sh‐*RCN1* (*n* = 3) (E, right). RCN1 reduction was confirmed by western blot analysis (E, right). One of the two independent experiments is shown in (E) and (F). One of the three independent experiments is shown in (G). *N* indicates the technical duplicates within one experiment of three independent replicates. Western blot images are representative of two experiments. Data are presented as the mean ± SEM. ^ns^ *P* > 0.05, **P < 0.01, ****P < 0.0001, as determined by unpaired two‐tailed Student's *t*‐test.

To investigate the role of RCN1 in AML cells, we utilized clustered regularly interspaced short palindromic repeats (CRISPR)/CRISPR‐associated protein 9 (Cas9) technology to establish three *RCN1* gene knockout cell clones (MOLM‐13‐23, MOLM‐13‐240, and MOLM‐13‐242) derived from the human AML cell line MOLM‐13. Western blotting confirmed a reduction in the level of RCN1 protein in these cell clones, which exhibited significantly reduced viability when compared to MOLM‐13 cells (Fig. 3A). Similarly, the viability of three other human AML cell lines (NB4, OCI/AML3, and THP‐1) was also significantly decreased following *RCN1* knockdown using a lentiviral shRNA vector (Fig. 3B–D). It is worth noting that downregulation of RCN1 did not affect the viability of several nontumor cell lines, including human embryonic kidney cells 293T/17 (Fig. 3E), human normal lung epithelial cells (BEAS‐2B), and human foreskin fibroblasts (HFF‐1; Fig. 3F). However, there is an exception: Human immortalized keratinocytes (HaCaT) exhibited a mild decrease in viability (Fig. 3G).

### 3.3 Downregulation of RCN1 induces the pyroptosis of AML cells by promoting IFN‐1 levels

**Fig. 4:**
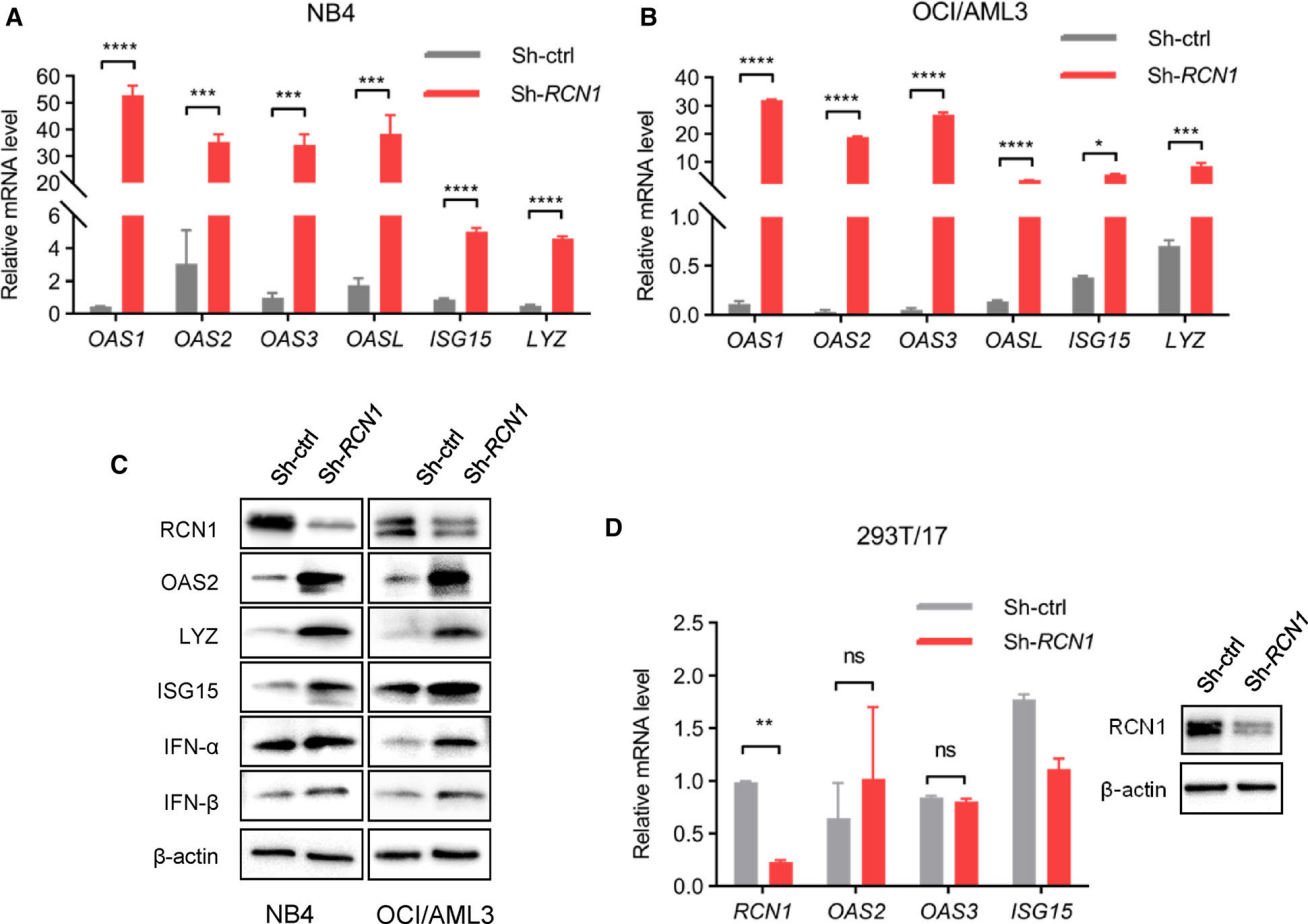
Downregulation of RCN1 induces the activation of IFN‐1 signaling in AML cells. (A, B) Relative mRNA levels of interferon‐stimulated genes (ISGs) in NB4 cells (A) and OCI/AML3 cells (B) were detected by qPCR, normalized to GADPH 4 days after being transfected with lentivirus sh‐ctrl (*n* = 3) or sh‐*RCN1* (*n* = 3). One of the three independent experiments is shown. (C) Relative protein levels of ISGs and IFN‐1 in OCI/AML3 (left) and NB4 (right) cells were detected by western blot 4 days after being transfected with lentivirus sh‐ctrl or sh‐*RCN1*. Western blot images are representative of three experiments. (D) Relative mRNA levels of *RCN1* and ISGs in 293T/17 cells were confirmed by qPCR 4 days after being transfected with lentivirus sh‐ctrl (*n* = 3) or sh‐*RCN1* (*n* = 3). Knockdown was confirmed by western blot. One of the two independent experiments is shown. *N* indicates the technical duplicates within one experiment of three independent replicates. Data are presented as the mean ± SD. ^ns^ *P* > 0.05, *P < 0.05, **P < 0.01, ***P < 0.001, ****P < 0.0001, as determined by unpaired two‐tailed Student's *t*‐test.

To determine the mechanism by which downregulation of RCN1 decreases the viability of AML cells, transcriptome analysis was performed on MOLM‐13‐23, MOLM‐13‐242, *RCN1*‐deficient NB4, and OCI/AML3 cells, along with their respective control cells. The Venn diagram showed that two genes consistently displayed differential expression in all four pairs of comparison samples (Fig. S2A). These genes were *LYZ* and *OAS3*, two ISGs that exhibited high expression in all four *RCN1*‐deficient cell lines (Fig. S2B). Additionally, qPCR results revealed higher expression levels of several other ISGs (*OAS1*, *OAS2*, *OAS3*, *OASL*, *ISG15*, and *LYZ*) in *RCN1*‐deficient NB4 cells (Fig. 4A) and *RCN1*‐deficient OCI/AML3 cells (Fig. 4B). Western blotting analysis also demonstrated increased levels of OAS2, ISG15, and LYZ proteins in *RCN1*‐deficient cell lines, compared with their corresponding control cells (Fig. 4C). Moreover, IFN‐α and IFN‐β levels were dramatically elevated in R*CN1*‐deficient NB4 and R*CN1*‐deficient OCI/AML3 cells (Fig. 4C). These data suggest that the reduction in cell viability could potentially be linked to the rise in IFN‐1 subsequent to the downregulation of RCN1. In contrast to AML cells, downregulation of RCN1 did not induce the expression of these ISGs in the nontumor cell line 293T cells (Fig. 4D), which may explain why these cells were not inhibited.

**Fig. 5:**
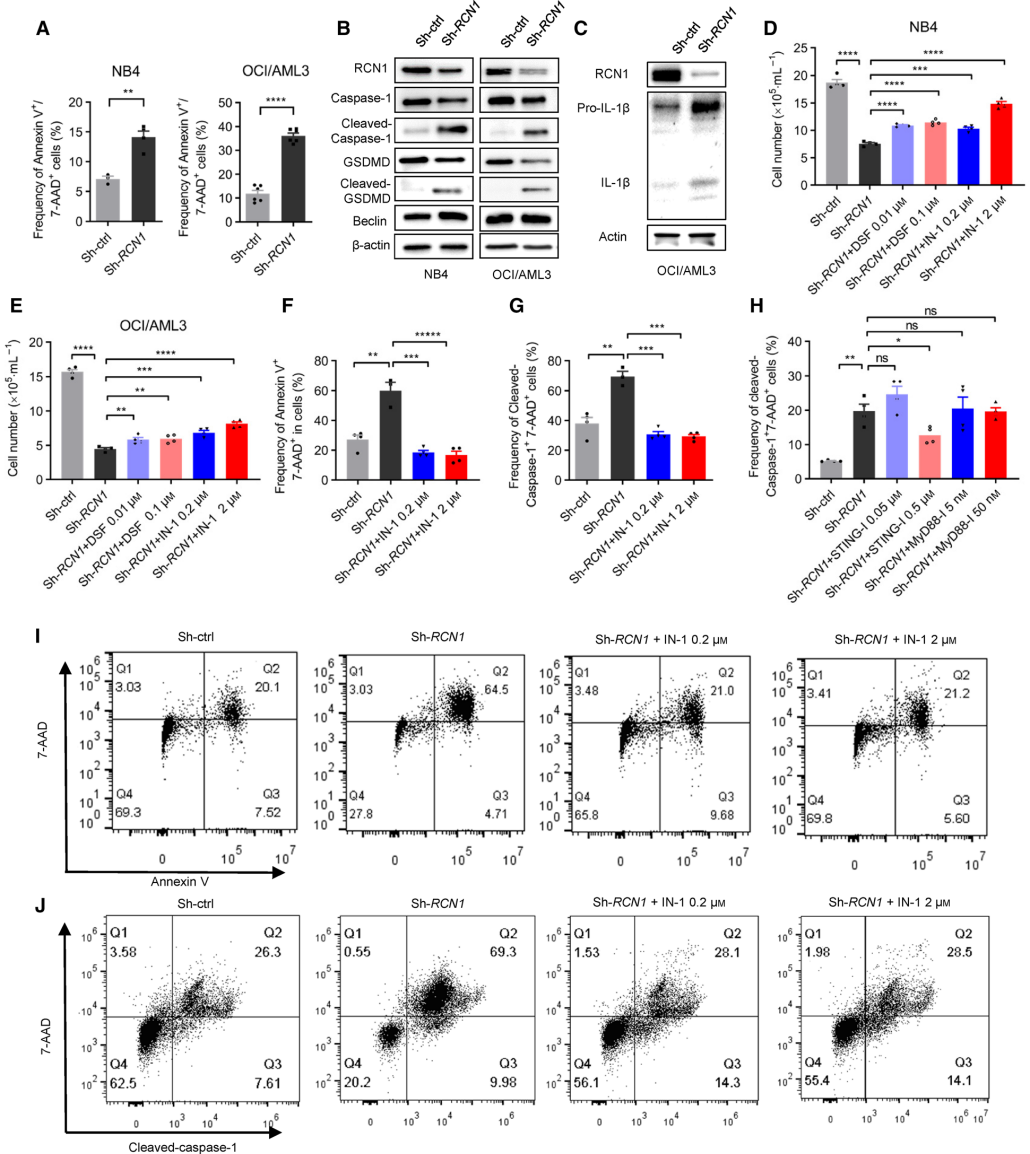
Reduction of RCN1 induces the pyroptosis of AML cells by activating IFN‐1 signaling. (A) The dead cells were detected by flow cytometry with Annexin V‐PE/7 AAD staining in NB4 (left; *n* = 3) 5 days or OCI/AML3 (right; *n* = 6) cells 7 days after being transfected with lentivirus sh‐ctrl or sh‐*RCN1*. One of the three independent experiments is shown. (B) Relative protein levels of pyroptosis‐related proteins and Beclin, an autophagy‐related protein in NB4 (left) and OCI/AML3 (right) cells were detected by western blots 7 days after transfection with lentivirus sh‐ctrl or sh‐*RCN1*. Western blot images are representative of three experiments. (C) Relative protein levels of pro‐IL‐1β and IL‐1β in OCI/AML3 cells were detected by western blots 4 days after transfection with lentivirus sh‐ctrl or sh‐*RCN1*. Western blot images are representative of two experiments. (D, E) The cell numbers of NB4 and OCI/AML3 cells were detected by cell count 7 days after transfection with lentivirus sh‐ctrl (*n* = 4) or sh‐*RCN1* (*n* = 4) and treated with the pyroptosis inhibitor disulfiram (DSF; *n* = 4), or the IFNαinhibitor IN‐1 (*n* = 4), or not (*n* = 4). One of the three independent experiments is shown. (F) Representative flow cytometric plots of Annexin V‐PE/7 AAD‐positive NB4 cells 7 days after being transfected with lentivirus sh‐ctrl (*n* = 4) or sh‐*RCN1* (*n* = 3) and treatment with IN‐1 (*n* = 4). One of the three independent experiments is shown. (G) The pyroptosis of NB4 cells was detected by the FLICA® 660 Caspase‐1 Assay Kit 5 days after transfection with lentivirus sh‐ctrl (*n* = 4) or sh‐*RCN1* (*n* = 3) and treated with IN‐1 (*n* = 4). One of the three independent experiments is shown. (H) The pyroptosis of NB4 cells was detected by the FLICA® 660 Caspase‐1 Assay Kit 6 days after transfection with lentivirus sh‐ctrl (*n* = 4) or sh‐*RCN1* (*n* = 4) and treated with inhibitors for STING (STING‐I; *n* = 4) and MyD88 (MyD88‐I; *n* = 4). One of the two representative experiments is shown. (I) Dot plots of flow cytometry data showing Annexin V‐PE/7 AAD‐positive cells in F. (J) Dot plots of flow cytometry data showing caspase‐1‐positive cells in G. *N* indicates the technical duplicates within one experiment of three independent replicates. Western blot images are representative of two experiments. Data are presented as the mean ± SEM. ^ns^ *P* > 0.05, *P < 0.05, **P < 0.01, ***P < 0.001, ****P < 0.0001, as determined by unpaired two‐tailed Student's *t*‐test.

Considering that IFN‐1 can induce cell pyroptosis, we hypothesize that pyroptosis could be one of the mechanisms by which downregulation of RCN1 decreases the viability of AML cells. We then used Annexin V‐PE/7AAD staining and flow cytometry to detect dead cells. We observed a significant increase in the proportion of Annexin V‐PE^+^/7AAD^+^ cells in *RCN1*‐deficient NB4 cells and *RCN1*‐deficient OCI/AML3 cells (Fig. 5A). IFN‐1 can induce pyroptosis via activated caspase‐1 and GSDMD, leading to activation of interleukin‐1b (IL‐1b) and IL‐18 [51, 52, 53]. Indeed, the cleaved‐caspase‐1 and cleaved‐GSDMD were increased in *RCN1*‐deficient NB4 cells and *RCN1*‐deficient OCI/AML3 cells when compared to the control cells, as determined by western blot analysis (Fig. 5B). As a negative control, Beclin, an autophagy‐related protein, was not affected by RCN1 downregulation (Fig. 5B). We have also conducted experiments to examine the cleavage of pro‐IL‐1β in *RCN1*‐deficient OCI/AML3 cells. The results showed that the IL‐1β was increased (Fig. 5C). To confirm the role of GSDMD, the *RCN1*‐deficient AML cells were treated with disulfiram (DSF), a specific inhibitor of acetaldehyde dehydrogenase type 1 (ALDH1) and effectively inhibits GSDMD pore formation. DSF treatment partially restored the decrease in NB4 and OCI/AML3 cell numbers caused by RCN1 downregulation (Fig. 5D,E). To address the role of IFN‐1, the *RCN1*‐deficient AML cells were treated with the IFN‐α inhibitor, IFN alpha‐IFNAR‐IN‐1 hydrochloride (IN‐1), and the decreased cell number of *RCN1*‐deficient NB4 and *RCN1*‐deficient OCI/AML3 cells was also restored (Fig. 5D,E). To further investigate whether the pyroptosis is caused by IFN‐1, we used cleaved‐caspase‐1/7AAD staining and flow cytometry to detect pyroptotic cells. The proportion of the Annexin V‐PE^+^/7AAD^+^ cells (Fig. 5F,I) and the cleaved‐caspase‐1^+^/7AAD^+^ cells (Fig. 5G,J) indicated that the dead and pyroptotic cells of *RCN1*‐deficient NB4 cells were reduced by IN‐1 treatment.

To assess the influence of the IFN‐1 production pathway on pyroptosis in *RCN1*‐deficient cells, we performed an experiment to examine the occurrence of pyroptosis in NB4 cells. This was accomplished by adding inhibitors for STING (STING‐I) and MyD88 (MyD88‐1). The proportion of cleaved‐caspase‐1^+^/7AAD^+^ cells indicated that the pyroptotic cells in *RCN1*‐deficient NB4 cells were restored by treatment with the STING inhibitor, but not by the MyD88 inhibitor (Fig. 5H). These results indicate that RCN1 affects pyroptosis through the DNA sensing pathway rather than the TLR pathway to control the production of IFN‐I in AML cells.

RCN1 has been identified as a potential participant in the cellular processes of endoplasmic reticulum (ER) stress and unfolded protein response (UPR) [19]. These processes may allow RCN1 to regulate intracellular apoptosis and pyroptosis. To investigate the impact of reducing RCN1 on ER stress and the UPR in AML cells, we assessed the levels of proteins associated with ER stress and the UPR. The results indicated that reducing RCN1 did not lead to a significant rise in the protein levels of GRP78, pPERK, peIF2a, and CHOP in NB4 or OCI/AML3 cells (Fig. S3A). Those results indicate that ER stress and the UPR were not activated following RCN1 downregulation in AML cells.

Taken together, those results suggest that downregulation of RCN1 promotes IFN‐1 levels, leading to the pyroptosis of AML cells.

### 3.4 Reduction of the *Rcn1* gene affects the viability of mouse AML cells but not mouse hematopoiesis

**Fig. 6:**
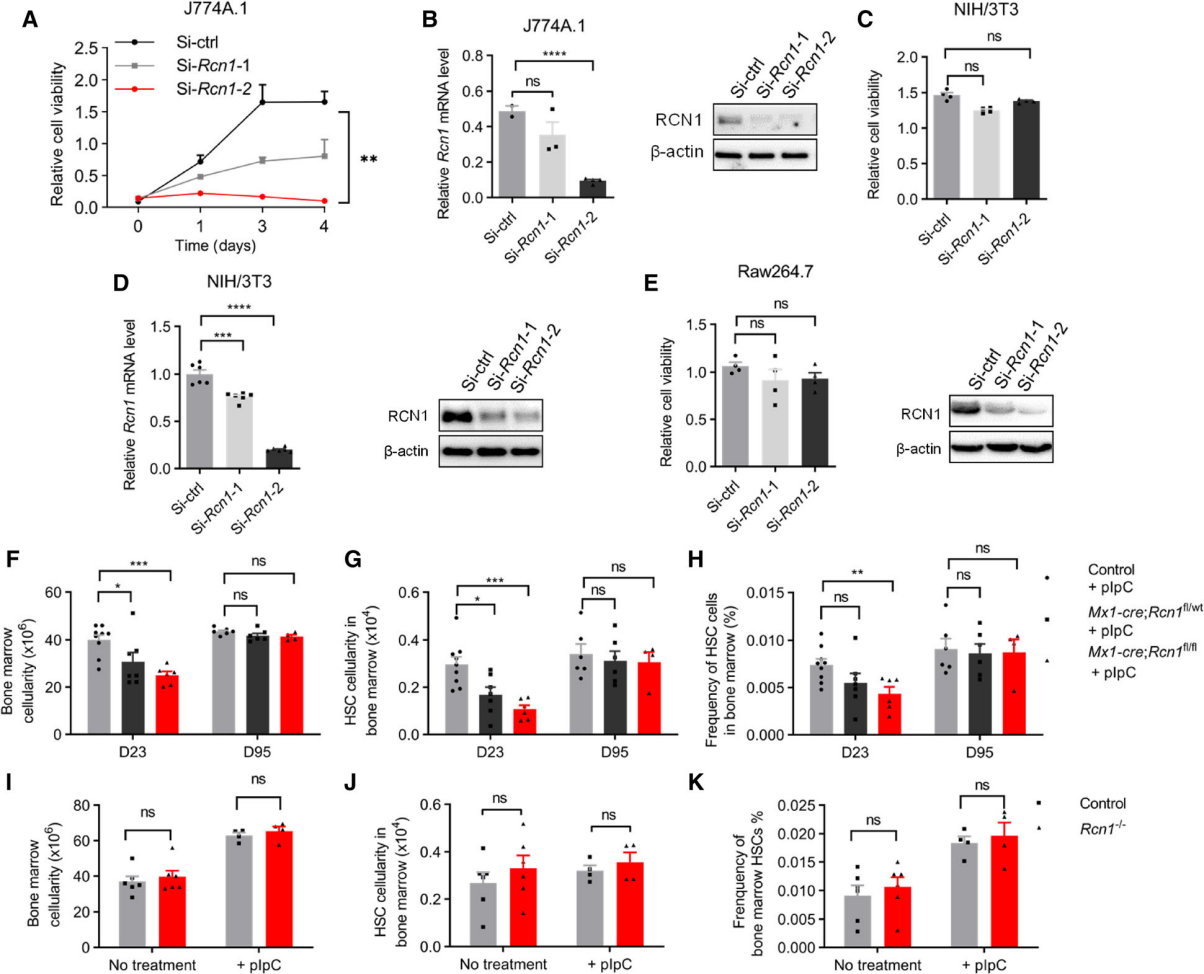
*Rcn1* gene deletion affects the growth of mouse AML cells but not mouse hematopoiesis. (A–E) Cell viability was detected by the CCK‐8 assay 7 days after being transfected with small interfering RNA (siRNA) si‐ctrl, si‐*Rcn1*‐1, or si‐*Rcn1*‐2 in J774A.1 cells (*n* = 6) (A), NIH/3T3 cells (*n* = 4) (C), and Raw264.7 cells (*n* = 4) (E, left). Relative *Rcn1* mRNA level was confirmed by qPCR in J774A.1 cells (Si‐ctrl, *n* = 2; si‐*Rcn1*‐1, *n* = 3; or si‐*Rcn1*‐2, *n* = 4) (B, left) and NIH/3 T3 cells (*n* = 6) (D, left). Relative Rcn1 protein level was confirmed by western blot in J774A.1 cells (B, right), NIH/3 T3 cells (D, right), and Raw264.7 cells (E, right). One of the three independent experiments is shown in (A, B). One of the two independent experiments is shown in (C–E). *N* indicates the technical duplicates within one experiment of three independent replicates. (F–H) The tibias and femurs of mice were harvested at the indicated time after pIpC treatment in controls, *Mx1‐cre*; *Rcn1* ^*fl*/*wt*^ or *Mx1‐cre*; *Rcn1* ^*fl*/*fl*^ mice, and then bone marrow cells were analyzed by flow cytometry or cell counting (Day 23, controls (*n* = 9), *Mx1‐cre*; *Rcn1* ^*fl*/*wt*^ (*n* = 7); *Mx1‐cre*; *Rcn1* ^*fl*/*fl*^ (*n* = 6); Day 95, controls (*n* = 6), *Mx1‐cre*; *Rcn1* ^*fl*/*wt*^ (*n* = 6); *Mx1‐cre*; *Rcn1* ^*fl*/*fl*^ (*n* = 4)). One of the two independent experiments is shown. (F) The cellularity of bone marrow cells. (G) HSC cellularity in the bone marrow. (H) Frequency of HSCs in the bone marrow. (I–K) Bone marrow cells were harvested in 2‐month‐old controls or *Rcn1* ^*−/−*^ mice 43 days after pIpC treatment or not. No treatment (controls (*n* = 6), *Rcn1* ^*−/−*^ (*n* = 6)); PIPC treatment (controls (*n* = 4), *Rcn1* ^*−/−*^ (*n* = 4)). (I) The bone marrow cellularity. (J) HSC cellularity in the bone marrow. (K) Frequency of HSCs in the bone marrow. One of the two independent experiments is shown. *N* indicates the number of samples. Data are presented as the mean ± SEM. ^ns^ *P* > 0.05, *P < 0.05, **P < 0.01, ***P < 0.001, ****P < 0.0001, as determined by unpaired two‐tailed Student's *t*‐test.

In order to carry out *in vivo* research and clarify the effect of *RCN1* on normal hematopoiesis, we first tested whether the homologous gene *Rcn1* has a similar effect on mouse AML cell lines. The SiRNA‐mediated knockdown of *Rcn1* significantly inhibited the viability of the mouse AML cell line J774A.1 (Fig. 6A,B). In contrast, *Rcn1* knockdown did not affect the viability of the mouse embryonic fibroblast cell line NIH/3T3 (Fig. 6C,D). Moreover, *Rcn1* knockdown did not decrease the viability of Raw264.7, another mouse AML cell line, which lacks the apoptosis‐associated speck‐like protein that contains a carboxyl‐terminal CARD (ASC) and, therefore, cannot activate caspase‐1 (Fig. 6E) [54].

To evaluate the impact of the *Rcn1* gene on mouse bone marrow hematopoiesis, we generated a floxed allele of *Rcn1* (*Rcn1* ^fl/fl^) (Fig. S4A) and crossed it with *Mx1‐cre* to generate *Mx1‐cre*; *Rcn1* ^fl/fl^ mice (Fig. S4B). *Rcn1* was conditionally deleted from the bone marrow hematopoietic cells by intraperitoneally injecting polyinosinic‐polycytidylic acid (pIpC) into 6‐ to 10‐week‐old *Mx1‐cre*; *Rcn1* ^fl/fl^ mice. The deletion of *Rcn1* was detected by PCR 10 days after the last pIpC injection (Fig. S4C). To test the influence of *Rcn1* deletion on mature blood cells, we did complete blood count (CBC) analyses. The CBC results revealed a decrease in the frequency of granulocyte cells and an increase in the frequency of lymphocyte cells in the peripheral blood of *Mx1‐cre*; *Rcn1* ^fl/fl^ mice compared with controls on Day 12 after the last pIpC injection (Fig. S5A,B). The proportion of monocyte cells and the red blood cell count (RBC) increased on Day 49 following the last pIpC injection (Fig. S5C,D). In addition, some minor changes were observed, including an increase in hemoglobin (HGB), hematocrit (HCT), mean platelet volume (MPV), and platelet distribution width (PDW) in *Mx1‐cre*; *Rcn1* ^fl/fl^ mice on Day 49 (Fig. S5G–J). All CBC parameters returned to normal on Day 94 after the last pIpC injection (Fig. S5A–J). In order to find out the cause of this phenotype, we analyzed the composition of bone marrow hematopoietic cells by flow cytometry (Fig. S6A). Compared with control mice, both *Mx1‐cre*; *Rcn1* ^fl/wt^ and *Mx1‐cre*; *Rcn1* ^fl/fl^ mice showed a significant decrease in bone marrow cellularity (Fig. 6F) and hematopoietic stem cells (HSCs) on Day 23 after the last pIpC injection (Fig. 6G). Additionally, the frequency of HSC cells was significantly lower in *Mx1‐cre*; *Rcn1* ^fl/fl^ mice on Day 23 after the last pIpC injection (Fig. 6H) while the frequencies of all detected hematopoietic progenitors and mature cells were unchanged in *Mx1‐cre*; *Rcn1* ^fl/fl^ mice compared with controls (Fig. S7A,B). Interestingly, the effect of *Rcn1* deletion was temporary. The cellularity of bone marrow and HSCs and the frequency of HSC cells returned to normal on Day 95 after the last pIpC injection (Fig. 6F–H). We also directly assessed the differentiation ability of bone marrow cells with *Rcn1* deletion by a CFUs assay. Bone marrow cells were isolated from *Mx1‐cre*; *Rcn1* ^fl/fl^, or control mice 7 days after the last pIpC injection and then plated in methylcellulose culture medium for 6 days, which supports myeloid‐erythroid differentiation *in vitro*. The CFUs were counted and photographed under an automated CFU assay reader. The results indicated that there was no difference in the number and size of colonies between the *Mx1‐cre*; *Rcn1* ^fl/fl^ groups and controls (Fig. S7C,D).

Considering the cell death caused by INF‐1 in RCN1 downregulation, we speculated that the transient effect of *Rcn1* deletion on HSCs might be due to the overlying of IFN‐1 signaling induced by pIpC. To exclude the interference of pIpC and to explore the effect of RCN1 on mice whole‐body, we crossed *Rcn1* ^fl/fl^ mice with *CMV‐cre* to generate *CMV‐cre*; *Rcn1* ^fl/fl^ mice with constitutive *Rcn1* deletion whole‐body, including germ cells (Fig. S4D). The offspring of *CMV‐cre*; *Rcn1* ^fl/fl^ mice, named *Rcn1* ^−/−^, had deleted *Rcn1* regardless of *CMV‐cre*. IFN‐α was detectable only in the plasma of pIpC‐treated *Rcn1* ^−/−^ mice (Fig. S8A). The CBC results showed that the PLT and RDW increased in *Rcn1* ^−/−^ mice on Day 20 and returned to normal on Day 32 after the last pIpC injection, but remained unchanged in *Rcn1* ^−/−^ mice without pIpC injection (Fig. S8G,H). PDW also had little change on Day 32 (Fig. S8I). No other abnormal CBC parameters had been observed (Fig. S8B–I). Furthermore, the number of bone marrow cells, the number of HSC cells, and the frequency of HSC cells in the bone marrow of *Rcn1* ^−/−^ mice did not change when compared to controls without pIpC treatment or with pIpC treatment after 43 days (Fig. 6I–K). Those data suggested that *Rcn1* deletion had no effect on hematopoiesis in the bone marrow of mice alone.

### 3.5 RCN1 is a potential target for human AML therapy

**Fig. 7:**
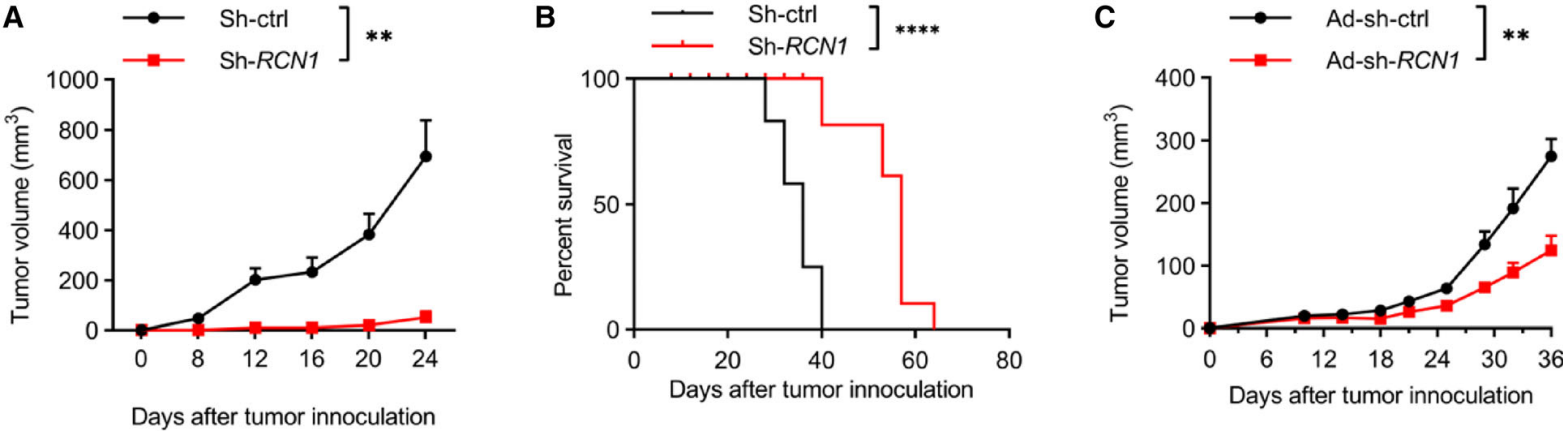
Downregulation of RCN1 inhibits the viability of AML *in vivo*. (A, B) 5 × 10^6^ THP‐1 cells transfected with the lentivirus sh‐ctrl (*n* = 10) or sh‐*RCN1* (*n* = 10) were inoculated into NCG mice subcutaneously in the right flank. The volume of tumors was measured every 4 days. One of the two independent experiments is shown. (B) The survival curve of NCG mice (A). (C) THP‐1 cells were inoculated into NSG mice subcutaneously on day 0.4 × 10^9^ Ad‐Sh‐*RCN1* (*n* = 6) or Ad‐Sh‐ctrl (*n* = 6) was intratumorally administered to mice on Day 4 and Day 7 after tumor inoculation. The volume of tumors was measured twice every week. *N* indicates the number of samples. One of the three independent experiments is shown. Unpaired *t*‐tests were used to analyze the data. The survival curves were generated by the Kaplan–Meier method and compared with the Gehan–Wilcoxon test. Data are presented as the mean ± SEM. ^ns^ *P* > 0.05, **P < 0.01, ****P < 0.0001.

To confirm the effect of downregulation of RCN1 on AML cells *in vivo*, we first used a murine xenograft model to evaluate the viability of *RCN1*‐deficient THP‐1 cells. The NCG mice inoculated with *RCN1*‐deficient THP‐1 cells had much smaller tumors and longer survival than those with control cells (Fig. 7A,B). We also established a murine xenograft model with THP‐1 cells in NSG mice. The tumor‐bearing NSG mice were treated with adenovirus targeting *RCN1* (Ad‐sh‐*RCN1*) or control adenovirus (Ad‐sh‐ctrl) on Day 4 and Day 7. The result indicated that downregulation of RCN1 will restrain the growth of human AML *in vivo* (Fig. 7C).

In all, knockdown of *RCN1* significantly reduced the viability of human AML both *in vitro* and *in vivo*, suggesting that it may be a promising target for AML therapy.

## 4 Discussion

RCN1 has been demonstrated to be highly expressed in a variety of tumor cells and is correlated with patient prognosis in renal cell carcinoma, prostate cancer, glioblastoma, non‐small cell lung cancer, oral squamous cell carcinoma, nasopharyngeal cancer, colon cancer, laryngeal cancer, highly aggressive breast cancer, sorafenib‐resistant hepatocellular carcinoma cells, and gemcitabine‐insensitive human pancreatic adenocarcinoma cells, among others [20, 21, 22, 24, 25, 27, 55, 56, 57, 58, 59, 60, 61, 62, 63]. Yoshida et al. [64] reported that RCN1 was expressed in lymphatic endothelial cells (T‐LECs) and lung cancer cells in lung tumors, but not in lymphatic endothelial cells (N‐LECs) in nontumor tissues. In breast cancer cells, RCN1 forms a complex with PIGX and RCN2 that negatively regulates the expression of ZIC family member 1 (ZIC1) and EH domain‐containing protein 2 (EHD2), promoting breast carcinogenesis [65]. Deletion of *RCN1* in prostate cancer cells significantly inhibits cell viability and leads to endoplasmic reticulum stress [27]. Several antitumor treatments can affect RCN1 expression. For example, 12‐o‐tetradecanoylphorbol‐13‐acetate (TPA) decreases RCN1 expression in the hypodifferentiated nasopharyngeal carcinoma squamous cell carcinoma cell line CNE2 cells [66], suberonylanilide hydroxamic acid (SAHA) decreases RCN1 expression in HepG2 cancer cells [67], and all‐trans retinoic acid significantly downregulates RCN1 expression in neuroblastoma SJ‐NK‐P cells [68]. Under hypoxic conditions, low‐dose γ‐irradiation promotes the survival of swelling epithelial cancer cells A431. During this process, the expression of calcium‐binding proteins CALM1, CALU, and RCN1 is increased [69]. Downregulation of these genes decreases the resistance of hypoxic tumor cells to low‐dose radiation. Additionally, the RCN1 content affects the sensitivity of cells to drugs. High RCN1 expression affects the sensitivity of hepatocellular carcinoma cells to sorafenib, while decreasing RCN1 content increases cellular sensitivity to antitumor drugs, such as adriamycin in uterine sarcoma cells and nasopharyngeal carcinoma cells [26, 55]. In one study, the opposite conclusion was reached, where increasing RCN1 protein levels in cisplatin‐resistant non‐small cell lung cancer with low RCN1 expression levels increased cell sensitivity to cisplatin [70]. Most of the mechanisms by which RCN1 affects tumors are unknown and require further investigation.

Our results showed that *RCN1* was relatively more highly expressed in the M3, M2, and M1 subgroups. Therefore, inhibiting the expression of *RCN1* in these groups may lead to improved treatment outcomes. And FLT3 mutations showed significantly higher levels of RCN1 expression compared with the wild‐type group. Combining FLT3 inhibitors with RCN1 inhibitors may be a new way to treat AML. We had evaluated the prognostic impact of RCN1 expression in AML patients from the TCGA and GTEx datasets (data not shown). The result showed that there was no significant difference in overall survival between the *RCN1* high expression group and the *RCN1* low expression group. We speculate that this may be related to the fact that the RCN1 high expression group is mainly associated with M3 types and FLT3 mutations, with M3 and FLT3 mutations having a good overall treatment outcome [71, 72], leading to an improved survival rate in the high RCN1 group. Despite the relative success of treatment for M3 and FLT3 mutations, relapse remains a major obstacle [73, 74]. The use of RCN1‐related inhibitors in combination with current treatments may offer a potential solution to address recurrence.

Acute myeloid leukemia (AML) is a disease that exhibits high heterogeneity and a poor prognosis, with a high rate of recurrence [75]. Recent studies have found that a pyroptosis‐related signature can effectively predict the prognosis of patients with AML [76, 77]. The process of inflammation‐related pyroptosis has emerged as a prospective target for cancer therapy [78]. The development of small‐molecule inhibitors of DPP8/9 that trigger pro‐caspase‐1‐dependent pyroptosis is a novel therapeutic approach with significant promise for treating AML [79].

In our study, downregulation of RCN1 promotes the IFN‐1 levels, leading to the pyroptosis of AML cells. Our results indicated that the decrement in RCN1 level in HEK293T/17 cells did not impact cell viability or activate ISG expression. Those results corroborate previous findings that established the incapacity of HEK293T/17 cells to activate IFN‐1 signaling due to the absence of STAT2 and IRF9 genes [80]. This may be the reason why the downregulation of RCN1 does not affect the viability of the HEK293T/17. Our data showed that the downregulation of RCN1 affects the viability of HaCaT cells, although it is not a tumor cell line. Previous studies have suggested that activation of interferon‐associated pathways can alter cell proliferation in HaCaT cells [81, 82]. We speculate that the decrease in HaCaT cell viability may be linked to the stimulation of interferon‐associated pathways.

On the contrary, we showed that RCN1 downregulation affects the viability of J774A.1 cells but not Raw264.7 cells. There is a difference between Raw264.7 cells and J774A.1 cells. J774A.1 cells can activate caspase‐1, while Raw264.7 cells cannot activate caspase‐1 because of the absence of an ASC [54]. All of our data on the human *RCN1* gene and mice *Rcn1* gene suggest that downregulation of RCN1‐induced cell pyroptosis may through elevating IFN‐1.

Based on the results of single‐cell RNA sequencing of primary AML cells, the frequency of Cluster 3 was reduced in the AML/M5‐si*R*C*N1* group compared with the control group. This cluster mainly expressed genes associated with cell viability. The downregulation of RCN1 was hypothesized to have affected the expression of viability‐related genes in AML cells, ultimately resulting in decreased cell proliferation and death. Our result showed that the proportion of Cluster 0 in the AML/M5‐si*RCN1* group was increased compared with the control group. This cluster mainly expresses genes related to leukocyte function. IFN‐1 can increase the proliferation and function of leukocyte cells [83, 84, 85, 86, 87]. We speculate that the reduction of RCN1 promoted IFN‐1 expression and thus enhanced the expression of leukocyte function‐related genes. A limitation of our study is that we need to see if IFN also regulates cell death via pyroptosis in AML patient samples, and we need to use more AML patient samples.

There are still many challenges in the treatment of AML. The emergence of numerous novel agents brings a new wave of hope for AML treatment. The objective for the future is to create novel drugs that can be combined with targeted medications or traditional chemotherapy to overcome drug resistance and toxicity [88]. Combining RCN1 inhibition with other treatments may provide a new way to address the toxicity and relapse problems of existing drugs.

## 5 Conclusions

Our results shed new light on AML treatments that can inhibit the viability of tumor cells without affecting normal cells. Our research reveals that RCN1 activates caspase‐1 and GSDMD to cause pyroptosis in AML cells via IFN‐1. Our study provides a new potential target for AML therapy.

## Conflict of interest

The authors declare no conflict of interest.

## Author contributions

SD was responsible for designing the review protocol, writing the protocol and report, conducting the experiments, extracting and analyzing data, and creating tables. YP was responsible for primary AML experiments, patient and healthy donor sample collection and analysis, and helping with mouse experiments. NA was responsible for performing colony‐forming unit assays and helping with mouse experiments. FC was responsible for performing complete blood count analyses. HC was responsible for the STING and MyD88 inhibitor assays. HW contributed to screening potentially eligible studies. XX and RL were responsible for extracting and analyzing ScRNA‐seq data. LY contributed to screening potentially eligible studies. XW contributed to providing feedback on the report and analyzing data. XD supervised this study, responsible for designing the review protocol and reviewing it critically for important intellectual content. QZ contributed to providing feedback on the report, designing the review protocol and screening potentially eligible studies, writing the report, extracting and analyzing data, and creating tables.

### Peer review

The peer review history for this article is available at https://www.webofscience.com/api/gateway/wos/peer‐review/10.1002/1878‐0261.13521.

## Supporting information


**Fig. S1.** scRNA‐seq data quality control.
**Fig. S2.** Transcriptome analysis of downregulated RCN1 in human AML cell lines.
**Fig. S3.** ER stress and the UPR were not activated following RCN1 downregulation in AML cells.
**Fig. S4.** Rcn1 is efficiently deleted from BMMC in Mx1‐cre; Rcn1fl/fl and Rcn1−/− mice.
**Fig. S5.** Rcn1 gene deletion in mouse bone marrow does not affect mature cells in the blood.
**Fig. S6.** Gating strategy.
**Fig. S7.** Deletion of Rcn1 gene has no impact on hematopoietic progenitors and mature cells.
**Fig. S8.** Complete blood count parameters of Rcn1 knockout mice.

**Table S1.** QPCR, PCR, and gene downregulation sequence.

**Table S2.** Abnormally expressed genes in the BMMC of AML patients (n = 5) compared with G‐CSF‐mobilized PBMC of healthy donors (n = 5).

**Table S3.** Participant information.

**Table S4.** Proportion of cluster per sample.

## Data Availability

The datasets used and/or analyzed and materials used during the current study are available from the corresponding author on reasonable request.
