# Supplementary material for: Downregulation of RCN1 promotes pyroptosis in acute myeloid leukemia cells
Source: Mol Oncol. 2023 Sep 30;17(12):2584–602. doi: 10.1002/1878-0261.13521 (PMC10701779; doi:10.1002/1878-0261.13521)
Supplement: Supplementary file 1 — Fig. S1. scRNA‐seq data quality control. Fig. S2. Transcriptome analysis of downregulated RCN1 in human AML cell lines. Fig. S3. ER stress and the UPR were not activated following RCN1 downregulation in AML cells. Fig. S4. Rcn1 is efficiently deleted from BMMC in Mx1‐cre; Rcn1fl/fl and Rcn1−/− mice. Fig. S5. Rcn1 gene deletion in mouse bone marrow does not affect mature cells in the blood. Fig. S6. Gating strategy. Fig. S7. Deletion of Rcn1 gene has no impact on hematopoietic progenitors and mature cells. Fig. S8. Complete blood count parameters of Rcn1 knockout mice. [file MOL2-17-2584-s002.pdf]

Supplementary Figures

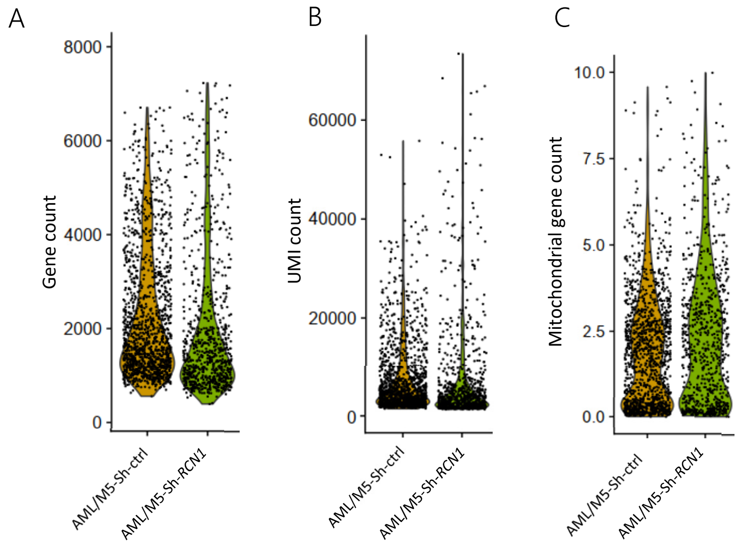

Fig. S1: scRNA-seq data quality control.

Violin plot for the distribution of (A) gene number, (B) UMI number, and (C) mitochondrial gene number per library.

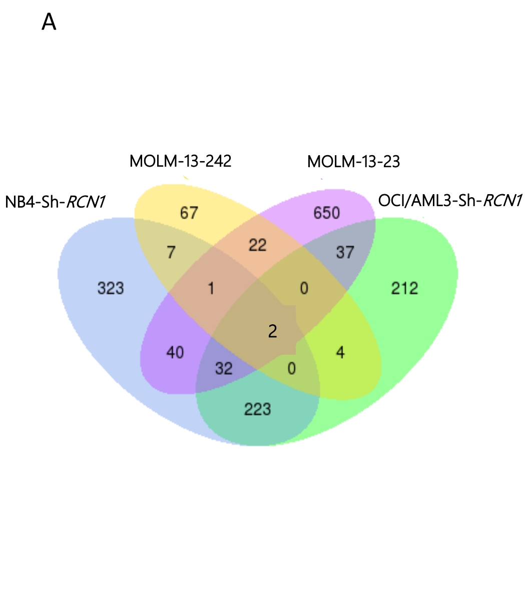

Fig. S2: Transcriptome analysis of downregulated *RCN1* in human AML cell lines. (A) Venn diagram for the overlap of differentially expressed genes from 4 pairs of samples with *RCN1* deficiency and control. (B) The transcripts per million (TPM) expression values of *LYZ* and *OAS3* gene in 4 pairs of samples.

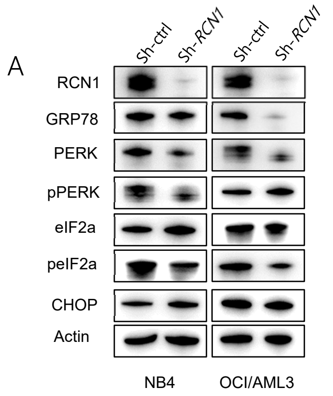

Fig. S3: ER stress and the UPR were not activated following RCN1 downregulation in AML cells.

(A) Relative protein levels of ER stress and unfolded protein response (UPR) in NB4 (left) and OCI/AML3 (right) cells were detected by western blots four days after transfected with lentivirus sh-ctrl or sh-*RCN1*.

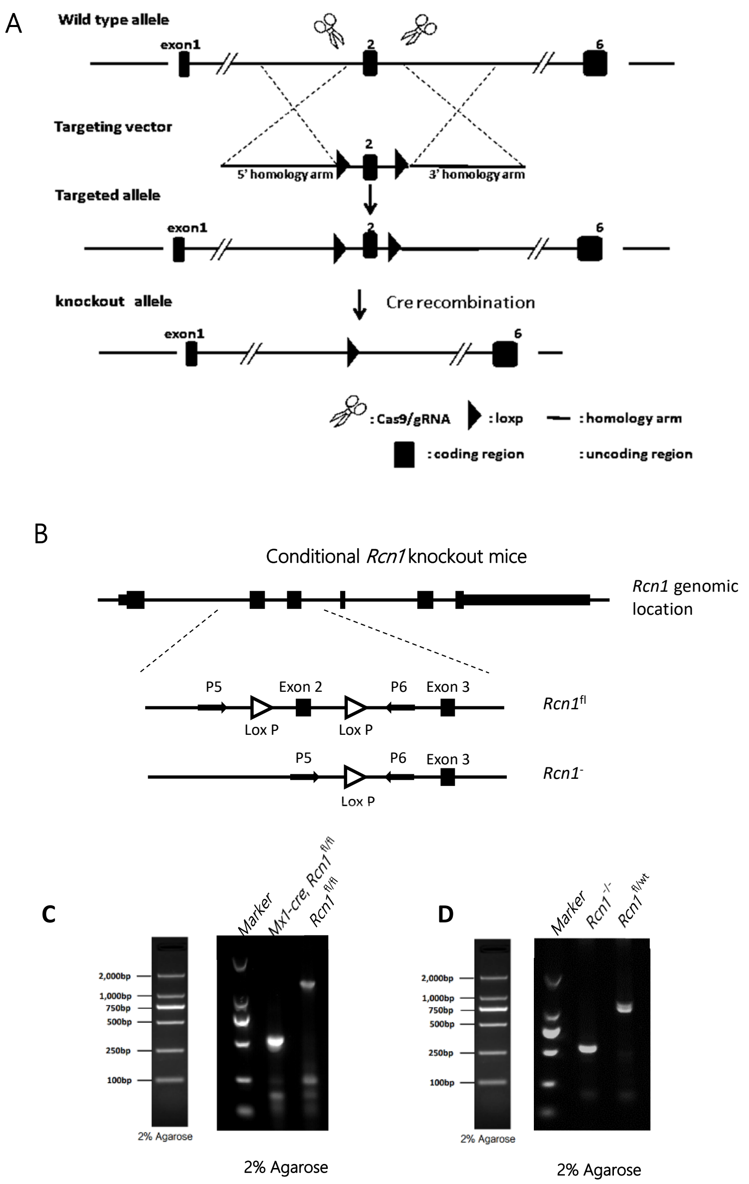

Fig. S4: *Rcn1* is efficiently deleted from BMMC in *Mx1*-cre; *Rcn1*$^{fl/fl}$ and Rcn1$^{-/-}$ mice.

(A) Using the homologous recombination principle and the method of homologous recombination in fertilized eggs, the Rcn1 gene was floxed modified. The brief process is as follows: Cas9 mRNA and gRNA were obtained by in vitro transcription. A homologous recombination vector (donor vector) was constructed using In-Fusion cloning, which contained a 3.0 kb 5' homologous arm, a 0.7 kb floxed region, and a 3.0 kb 3' homologous arm. Cas9 mRNA, gRNA, and donor vector were microinjected into fertilized eggs of C57BL/6J mice to obtain F0 generation mice. And F0 generation mice

were then bred with C57BL/6J mice to obtain a stable genetic F1 generation of mice. (B) Schematic of Cre-mediated recombination of the *Rcn1*^{fl} allele. The deletion of *Rcn1* was detected ten days after the last pIpC injection of *MX1-cre*; *Rcn1* $^{fl/fl}$ (C) and Rcn1$^{-/-}$(D) by PCR with primers P5 and P6.

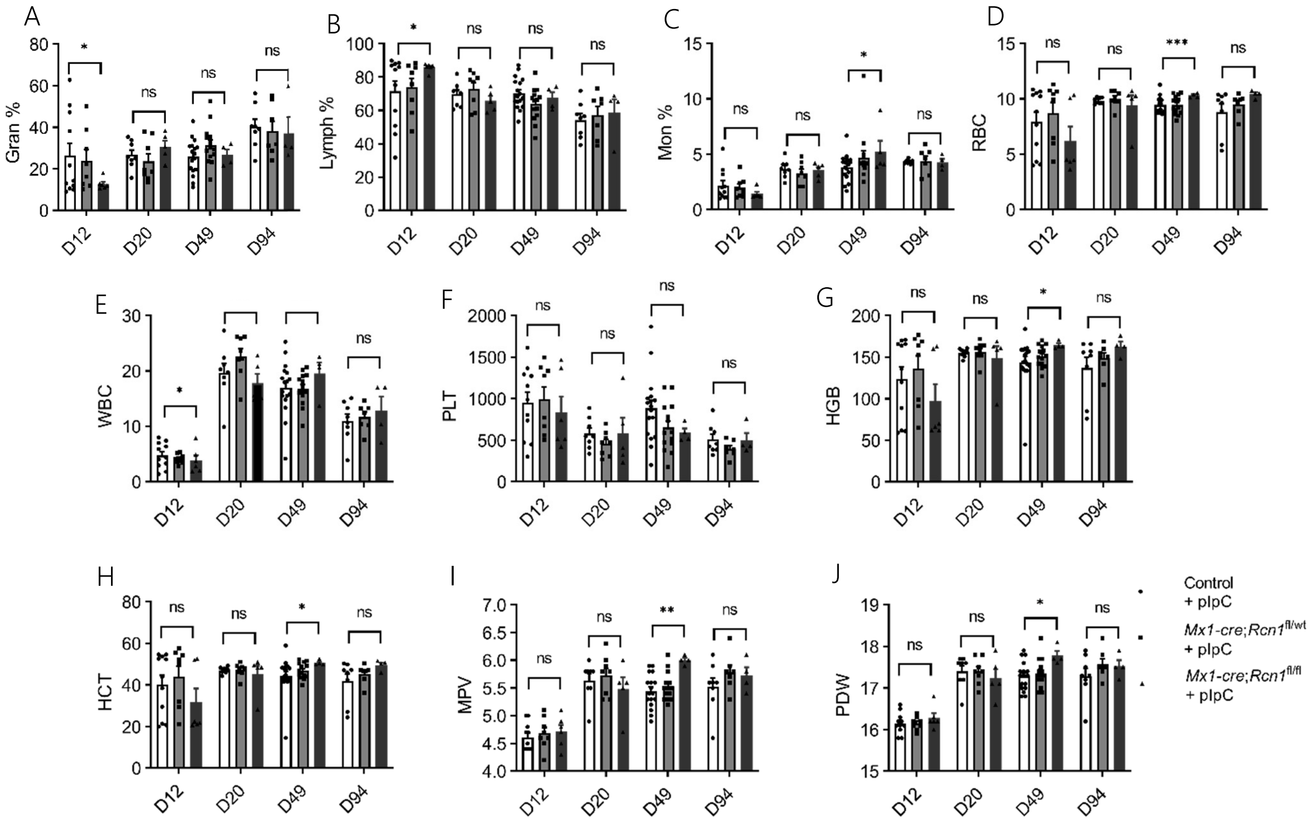

Fig. S5: *Rcn1* gene deletion in mouse bone marrow does not affect mature cells in the blood.

(A) Parameters of complete blood count (CBC) analyses on controls, *Mx1-cre*; *Rcn1*$^{fl/wt}$ mice and *Mx1-cre*; *Rcn1*$^{fl/fl}$ mice as indicated times after the last pIpC treatment (Day12, n=6-11; Day 20, n=5-8; Day 49, n=4-17; Day 94, n=4-8). (A)The frequency of granulocyte cells (Gran %). (B)The frequency of lymph cells (Lymph %). (C) Frequency of monocyte cells (Mon %). (D) Red blood cell count (RBC). (E) White blood cell count (WBC). (F) Platelet count (PLT). (G) Hemoglobin (HGB). (H) Hematocrit (HCT). (I) Mean platelet volume (MPV). (J) Platelet distribution width (PDW). Data are presented as the mean ± SEM. Ns P>0.05, *P<0.05, **P<0.01, ***P<0.001, as determined by unpaired two-tailed Student's t-test.

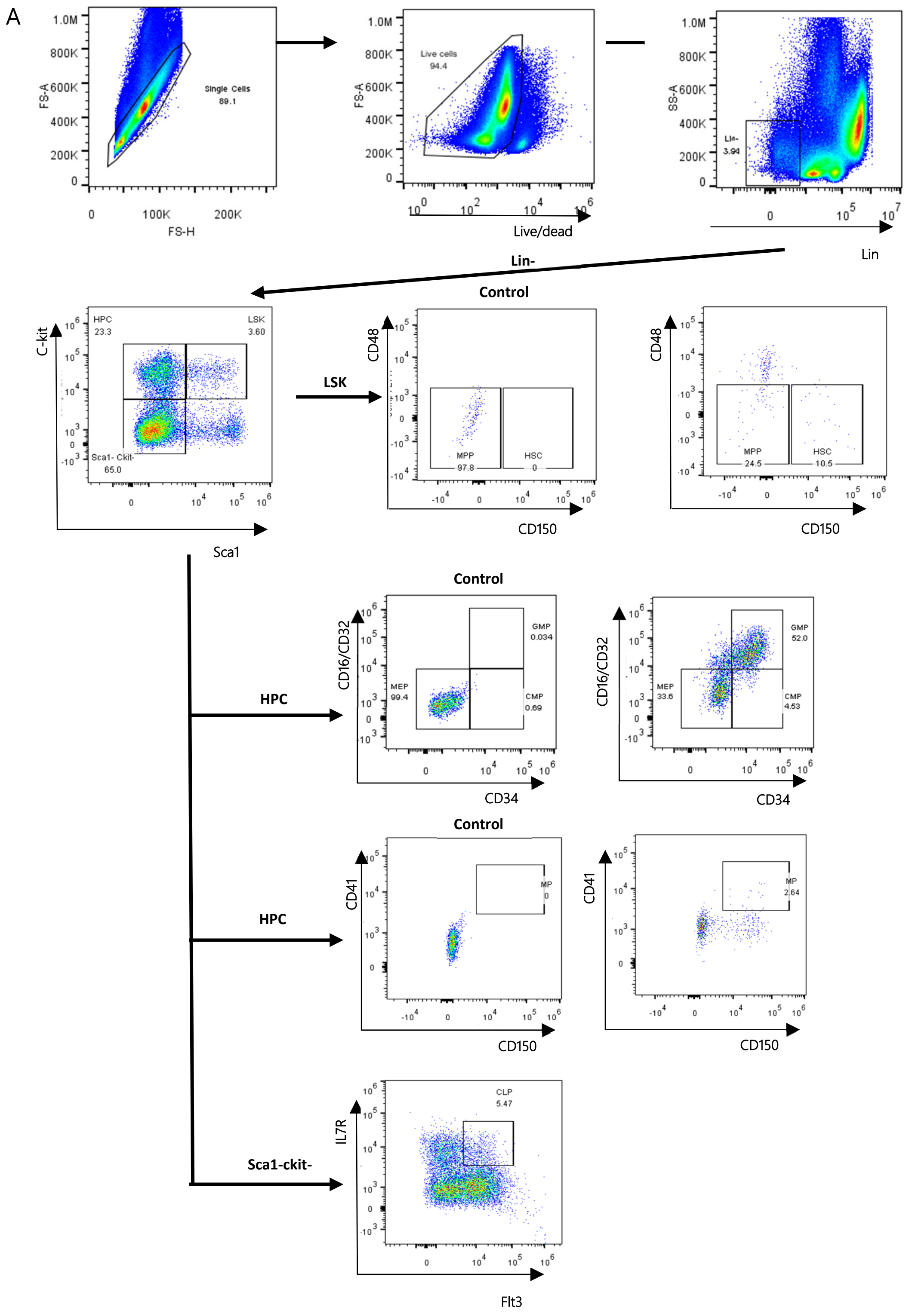

Fig. S6: Gating strategy.

(A) Representative flow cytometric plots showing the gating strategy used to identify HSCs in the bone marrow of mice.

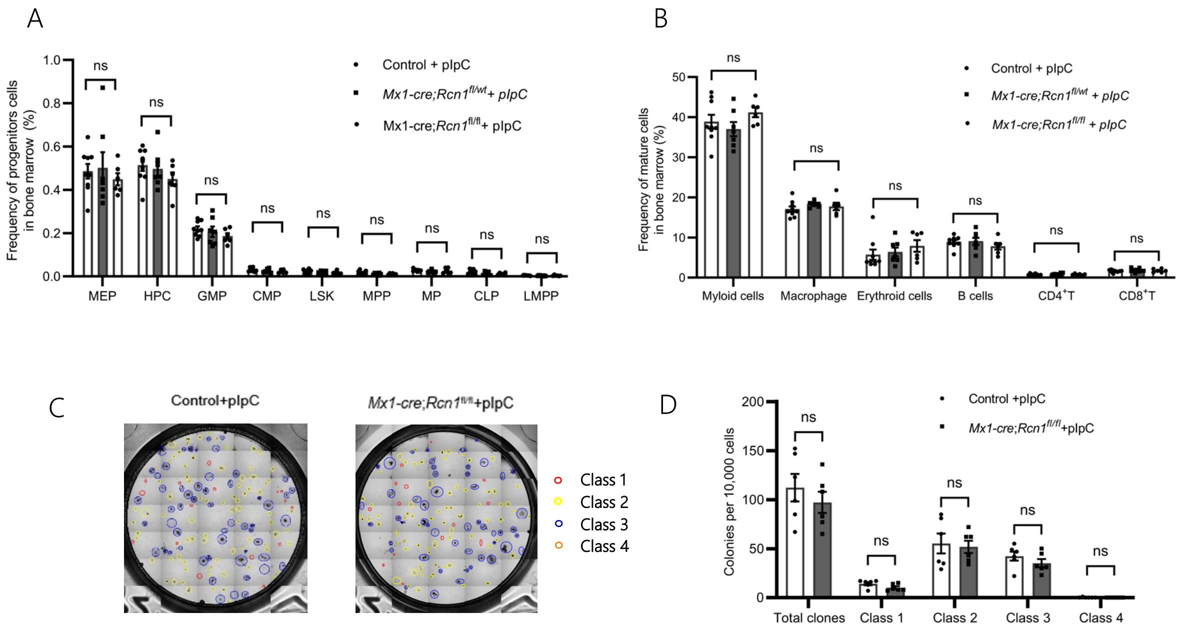

Fig. S7: The deletion of *Rcn1* gene has no impact on hematopoietic progenitors and mature cells.

(A and B) Frequency of hematopoietic progenitors (A) and mature cells (B) in bone marrow 23 days after the last pIpC treatment (n=10 for control, n=8 for *Mx1-cre*; *Rcn1*$^{fl/wt}$, n=4 for *Mx1-cre*; *Rcn1*$^{fl/fl}$). One of the two representative experiments is shown. (C) Representative image of clones from control (left) and *Mx1-cre*; *Rcn1*$^{fl/fl}$ (right) BMMC seven days after the last pIpC injection. (D) A number of colonies formed from 10,000 bone marrow cells (n=6 for both control and *Mx1-cre*; *Rcn1*$^{fl/fl}$). Data are presented as the mean ± SEM. Ns P>0.05 as determined by unpaired two-tailed Student's t-test.

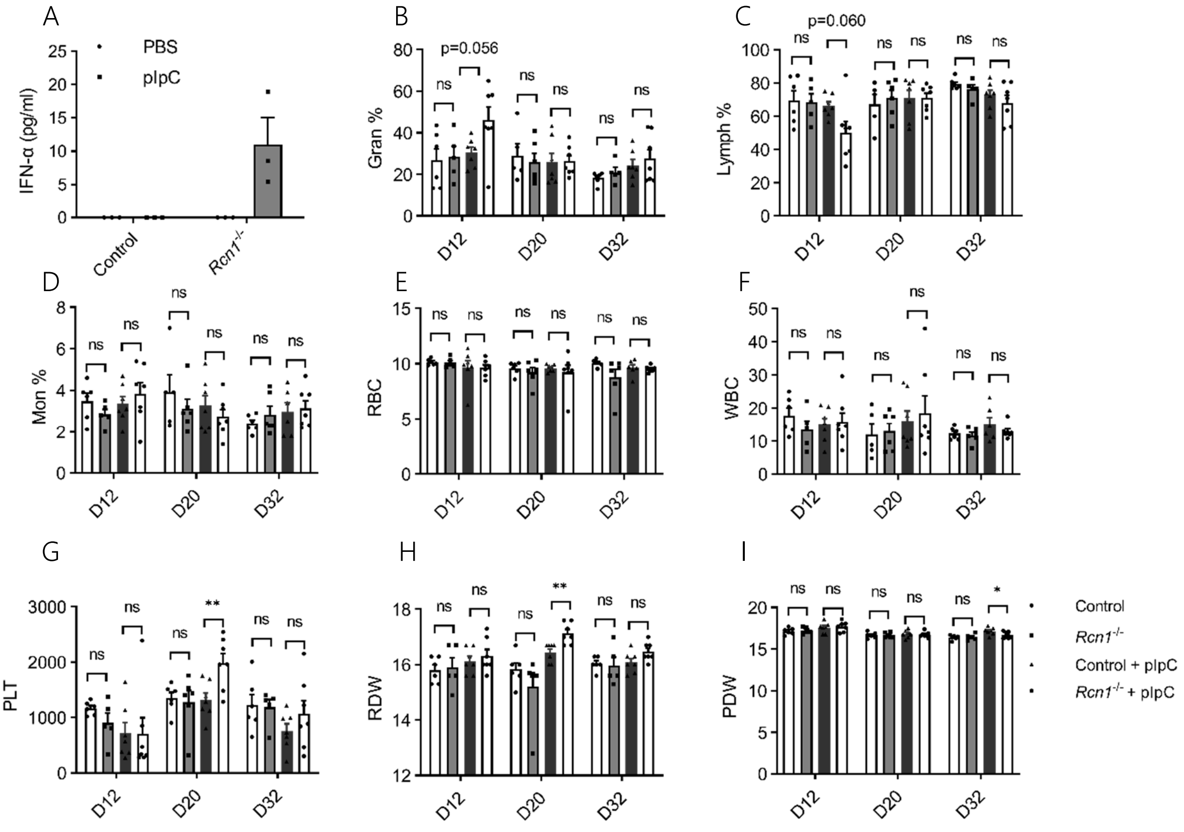

Fig. S8: Complete blood count parameters of *Rcn1* knockout mice.

(A) IFN-α concentration in the blood plasma of control and *Rcn1*$^{-/-}$ mice with pIpC treatment or not. 0 means underdetermination. (B-I) Parameters of complete blood count on control and *Rcn1*$^{-/-}$ mice after pIpC treatment or not as indicated time after the last treatment (n=7). (B) The frequency of granulocyte cells (Gran %). (C) The frequency of lymphocytes cells (Lymph %). (D) The frequency of monocyte cells (Mon %). (E) Red blood cell count (RBC). (F) White blood cell count (WBC). (G) Platelet count (PLT). (H) Red blood cell distribution width (RDW). (I) Platelet distribution width (PDW). Data are presented as the mean ± SEM. Ns P>0.05, *P<0.05, **P<0.01, as determined by unpaired two-tailed Student's t-test.
